# Spatial and Temporal Analysis of Fluorine Mass Balance in Swedish Sludge and the Impact of Sample Treatment on Extractable Organofluorine

**DOI:** 10.1021/acs.est.6c03578

**Published:** 2026-07-21

**Authors:** Felicia Fredriksson, Ulrika Eriksson, Anna Kärrman, Leo WY Yeung

**Affiliations:** Man-Technology-Environment (MTM) Research Centre, School of Science and Technology, 6233 Örebro University, Örebro SE-701 82, Sweden

**Keywords:** per- and polyfluoroalkyl substances (PFAS), sludge, extractable organofluorine, fluorine mass balance, side-chain fluorinated copolymers

## Abstract

Per- and polyfluoroalkyl substances (PFAS) are an extensive group of anthropogenic compounds with a large diversity of classes. Relying only on target analysis may underestimate the level of PFAS contamination in the environment. The extractable organofluorine (EOF) mass balance is a widely applied method to estimate the fraction of organofluorine not captured by target analysis. This study aimed to conduct a comprehensive EOF mass balance analysis on sludge and evaluate the impact of three different sample treatments on target PFAS and EOF, as well as their respective mass balance. Two complementary sample treatments were applied to 26 Swedish sludge samples: ion-pair extraction (IPE) for anionic and neutral PFAS, and hexane:acetone extraction (Hx:Ac) for nonpolar PFAS. The results demonstrated that the EOF concentrations were highly influenced by the sample treatment. The IPE method observed up to 40 times higher EOF concentrations than the Hx:Ac method, indicating a high proportion of polar organofluorine. Unidentified EOF increased between 2004 and 2017 for both sample treatments. These findings demonstrate that both polar and nonpolar organofluorine contribute to the EOF and that no single method captures them. In the present study, only two side-chain fluorinated copolymers were monitored, and other fluorinated polymers may be contributors to the unknown EOF.

## Graphical abstract

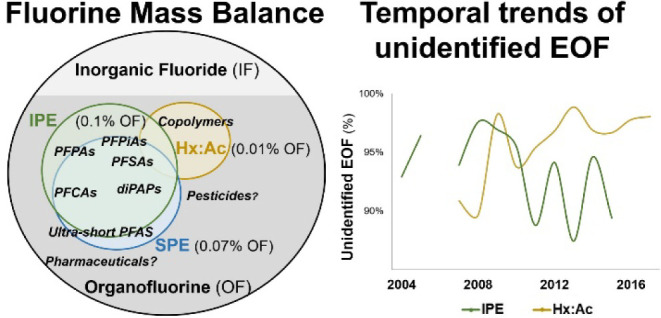

## Introduction

Wastewater treatment plants (WWTPs) have been recognized as substantial concentrators of per- and polyfluoroalkyl substances (PFAS) in the environment.^1–5^ PFAS entering WWTPs originate from a wide range of industrial and commercial applications. Besides the direct emissions of known PFAS, an increase of perfluoroalkyl carboxylic acids (PFCAs) and perfluoroalkyl sulfonic acids (PFSAs) concentrations in wastewater effluent compared to influent samples has been observed.^6–8^ One of the reasons might be the degradation and transformation of precursor compounds during wastewater treatment processes. Sludge has been pointed out to be a potential pathway for PFAS to enter the environment through the application of sludge on farmlands.^9,10^ In Sweden, thousands of tons of sludge are produced yearly. In 2020, sludge production was estimated at approximately 200 000 tons, and agricultural use was one of the most common sludge management practices, accounting for 46% of the total sludge production.^11^ Other ways to handle sludge include land application (23%), landfill coverage (16%), and incineration (2.5%).

Thousands of PFAS exist on the global market, and the largest share of PFAS are polymeric PFAS,^12^ with one of the classes included being the side-chain fluorinated polymers. Side-chain fluorinated polymers have been observed to be potential precursors to other nonpolymeric PFAS.^13–19^ Our recent study, together with a previous one, showed a detection frequency of 100% for fluoroalkyl sulfonamide (FASA)-based copolymers in Swedish sludge^20^ and Canadian biosolids,^21^ which demonstrates their widespread occurrence and the importance of monitoring this class of PFAS. Even if there are thousands of PFAS, only a few of them, such as PFSAs and PFCAs, are targeted in environmental monitoring studies because of limitations and challenges in extracting and analyzing PFAS, especially polymeric PFAS. For example, commonly used extraction methods for PFAS may not be able to capture the polymers.^20–23^

New PFASs are being introduced into the global market, which makes it even more difficult to obtain a holistic overview of the occurrence of PFASs. Thorough risk assessments of WWTPs require measurements of not only the legacy PFAS but also the total amount of PFAS released; otherwise, an underestimation of PFAS contamination via WWTPs may occur. Target analysis is limited in its capacity to measure the total amount of PFAS due to the lack of analytical standards and the unknown identities of PFAS, while nontarget analysis provides qualitative information only. To overcome this issue, several methods such as adsorbable organic fluorine (AOF), oxidative conversion (also known as the total oxidizable precursor (TOP) assay), particle-induced gamma-ray emissions (PIGE), and extractable organofluorine (EOF) have been developed and applied to various matrices, which allow for a more comprehensive PFAS analysis, including unknown PFAS and possible precursors to PFSAs and PFCAs.^24–28^ A fluorine mass balance can be conducted by quantitative measurements of total fluorine (TF) and free inorganic fluoride (IF).^29^ A proportion of the organofluorine (OF) within the TF can be extracted and is referred to as EOF. This additional extraction step isolates the organofluorine fraction, which is generally considered to be anthropogenic and largely associated with PFAS. Several studies have reported that only a minor fraction of EOF is explained by the target PFAS, suggesting that a large quantity of the EOF is unidentified.^30–33^ The unidentified fraction of the EOF may consist of nontarget PFAS, as the number of target compounds included in analytical methods varies substantially between studies, ranging from tens to hundreds of analytes, and even targeted methods do not comprehensively cover all PFCAs and PFSAs. Furthermore, other classes of organofluorines, such as pharmaceuticals, agrochemicals, and fluorinated polymers, may also contribute to the unidentified fractions. Research on the use of the EOF mass balance approach has increased; however, comparing and interpreting results remain challenging, as EOF results are highly dependent on sample treatment. This is an inherent consideration in EOF and other nontarget or summative analytical approaches performed without the use of mass-labeled internal standards.^29–34^ In order to understand more about the characteristics and identity of organofluorine, several extraction methods need to be tested to understand the extraction efficiency for different types of organofluorine.

The overall aim of this study was to obtain a more realistic picture of the environmental impact of PFAS in sludge, since currently, a significant proportion may be hidden by limitations in extraction methods. This was gained by conducting a comprehensive EOF analysis as a proxy for total PFAS and a mass balance analysis of organofluorine in sludge. The objectives were: (i) to evaluate the impact of three different sample treatments on target PFAS contribution to EOF and the relationship between EOF, OF, free IF, and TF in sludge; (ii) to assess the levels of unidentified EOF in sludge from Swedish WWTPs using two selected complementary sample treatments; and (iii) to provide more insight into a comprehensive EOF analysis and mass balance analysis of organofluorine using sludge from different WWTPs (regional differences) and from the years 2004 to 2017 (temporal variation).

## Materials and Methods

### Sample Collection

A standard reference material (SRM) from the National Institute of Standards and Technology (NIST), 2781 domestic sludge,^35^ referred to as US sludge, was used together with a pooled sludge sample (SWE). Freeze-dried Swedish sludge samples from different years, WWTPs, and amounts were pooled and homogenized together to maximize PFAS class diversity. The sludge samples were collected as part of the long-term national monitoring program by the Swedish Environmental Protection Agency.

Sludge samples (*n =* 26) included in this study originated from four different WWTPs located in Sweden (Borås, Stockholm, Torsås, and Umeå). More detailed information about the four WWTPs can be found in our previous study,^20^ where all samples were analyzed for target PFAS. These samples were re-extracted and analyzed for EOF in the present study. In brief, the Swedish Environmental Protection Agency collected all samples in October during a long-term national monitoring program. Sludge samples from Borås and Stockholm WWTPs were obtained between 2004 and 2017, excluding 2006 and 2016. In 2015, two additional sludge samples from Torsås and Umeå WWTPs were used to compare the EOF concentrations between WWTPs. All sludge samples were freeze-dried and stored at −18 °C prior to analysis.

### Chemicals and Materials

Detailed information about target analytes, standards, solvents, and materials is provided in the Supporting Information (SI).

### Sample Treatment

The three sample treatments chosen included two commonly used in EOF mass balance research, described to capture organofluorine of the anionic and neutral PFAS:^29^ (i) alkaline digestion followed by an adapted ion-pair extraction, and (ii) alkaline digestion followed by a modified solid-phase extraction (SPE) in order to remove the inorganic fluoride. The third and last sample treatment chosen was (iii) the Hx:Ac extraction method, customized for extracting nonpolar PFAS, including the two FASA-based copolymers. Briefly, for sample treatments i and ii, sludge samples were pretreated by alkaline digestion (1 M sodium hydroxide in methanol) and subsequently extracted using methanol. The extracts were subsequently purified either by ion-pair extraction (i) or SPE (ii). In the ion-pair method, aqueous tetrabutylammonium bisulfate (TBA) solution and methyl *tert*-butyl ether were used to separate organic fluorine into the organic phase, while inorganic fluoride remained in the aqueous phase. In the SPE method, the extract was diluted to 95% water and purified using an Oasis weak anion exchange cartridge, following ISO method ISO/DIS 21675, with additional washing steps to remove the inorganic fluoride. For sample treatment iii, sludge samples were extracted with acetone:hexane (1:1) and purified using silica-based SPE, followed by dispersive SPE with Supel QuE Z-Sep sorbent to remove the inorganic fluoride. The sample treatments are fully described in the SI.

### Instrumental Analysis

The sludge extracts were separately analyzed for EOF and a broad range of PFAS (*n* = 85), including the C4- and C8-FASA-based copolymers. Primarily, the PFAS analysis was performed on an Acquity Ultra Performance Liquid Chromatograph (UPLC) system coupled to a XEVO TQ-S tandem mass spectrometer (MS/MS; Waters Corporation, Milford, USA), in negative electrospray ionization mode. The analytical methods are fully described in the SI, Tables S1 and S3.

The EOF and TF concentrations were determined using a Combustion Ion Chromatograph (CIC) consisting of a combustion module (Analytik Jena, Germany), a 920 absorbent module, and a 930 Compact IC Flex (Metrohm, Switzerland). The separation of anions was performed on an ion-exchange column (Metrosep A Supp 5, 4 mm × 150 mm) with isocratic elution (64 mM sodium carbonate [Na_2_CO_3_] and 20 mM sodium bicarbonate [NaHCO_3_], after 20 times dilution). In brief, 100 μL of the extract or the solid sample (0.01 ± 0.0002 g) was placed on a quartz glass boat and combusted with hydropyrolysis at 1050 °C. During combustion, all fluorine was converted into hydrogen fluoride (HF) and was absorbed into ultrapure water (18.2 MΩ). The levels of fluoride ions in the solution were analyzed using ion chromatography and conductivity detection.

The IF experiment was conducted using a leaching method with ultrapure water (18.2 MΩ). Ten mL of ultrapure water was added to 0.02 or 1.00 g of freeze-dried sludge, and the mixture was shaken for 24 h. The sample was then centrifuged for 10 min at 8000 *g*, and the supernatant was filtered (0.2 μm GHP filter; Waters Corporation) and transferred to a new polypropylene tube. The concentrations of dissolved IF in the recovered supernatant were determined using ion chromatography (IC; Metrohm IC using Thermo IonPac AG12 and AS12A 4 × 200 mm columns) equipped with a conductivity detector. Mobile phases were 2.7 mM Na_2_CO_3_/0.3 mM NaHCO_3_ in isocratic elution at a 1.0 mL/min flow rate.

### Fluorine Mass Balance

The fluorine mass balance is composed of all fluorinated compounds in a neat sample and can be of both organic and inorganic origin. The total fluorine was obtained for the US and the pooled SWE sludge samples by combustion in the CIC without any pretreatment. The total fluorine consists of extractable organofluorine determined by the CIC, dissolved inorganic fluoride determined by IC, and nonextractable fluorine.

### Extractable Organofluorine Mass Balance

The extractable organofluorine mass balance was calculated by converting all quantifiable PFAS concentrations (ng/g) to the corresponding fluoride concentration (ng/g F). The conversion was done using the following equation:

$$c_{F}=\frac{n_{F}MW_{F}}{MW_{PFAS}}\times c_{PFAS}$$

where *C_F_* is the corresponding fluoride concentration (ng/g F), *n_F_* stands for the number of fluorine atoms in the PFAS, *MW_F_* is the molecular weight of fluorine, *MW_PFAS_* stands for the molecular weight of PFAS, and *C_PFAS_* is the quantifiable PFAS concentration determined using UPLC-MS/MS. Another approach was used for converting C8- and C4-FASA-based copolymers into fluoride equivalents, as the chemical structures of these compounds are not known. The fluoride content (%) of the technical mixtures was determined using CIC; the conversion was then made by multiplying the determined fluoride content (%) of the technical mixtures (used as a native standard) with the quantifiable concentrations (ng/g d.w.) of the C8-based and C4-based components measured in the samples by UPLC-MS/MS.

### Quality Assurance and Quality Control

Detailed information about quality assurance and quality control measurements is provided in the SI. For quantification of target analytes, mass-labeled internal standards were added to the sample extract after sample treatment, and quantification was performed using isotope dilution. Recoveries of the mass-labeled internal standards were within 75–125%. For the analytes for which no isotopically labeled standards were available, the homologue closest in retention time was used for quantification. For perfluorinated phosphonates (PFPAs) and perfluorinated phosphinates (PFPiAs), no mass-labeled internal standards were available, and quantification was performed using a matrix-matched calibration curve. Quantification of the FASA-based copolymers was done with a three-point standard addition method to account for ionization effects. The TF and EOF results were obtained using a five-point external calibration curve (50 to 1000 ng/mL F) made from a solid PFOA potassium salt (Fluka, Hampton, United States).

Quantification of fluoride was performed by subtracting the peak area of fluoride in the combustion blanks from that in the samples and applying a five-point external calibration curve (50 to 1000 ng/mL) for calculation. Procedure blanks were included in each batch and processed using the same extraction procedures as the sludge samples. The limits of detection (LOD) for EOF (40 to 70 ng/g dw) and target analytes (0.08 to 20 ng/g dw) were defined as the average blank concentration plus three times the standard deviation. When analytes were not detected in the procedure blanks, the lowest calibration point was used as the LOD (Tables S5–S7). Duplicate injections were conducted for dissolved IF, and the concentrations were reported as the average of the injections. Quantification of dissolved IF was conducted using a five-point calibration curve (0.1 to 10 mg/L) with a multistandard calibration solution containing F^–^, Cl^–^, NO_2_^–^, NO_3_^–^, Br^–^, PO_4_^3–^ , SO_4_^2–^ prepared by dilution and mixing of stock solutions (1 g/L) with ultrapure water (18.2 MΩ). The lowest calibration point was used as the detection limit. Stock solutions were prepared from analytical-grade sodium salts, and their concentrations after mixing were verified by analysis of the Fluka 89886 Certified Multi-Standard Solution. Calibration blanks contained ultrapure water and were freshly prepared before each calibration.

## Results and Discussion

### The Fluorine Types in Sludge and Sample Treatments’ Influence on EOF

To assess the relationship between EOF, free IF, and TF in sludge and evaluate the impact three different sample treatments have on EOF and target PFAS, a comprehensive workflow was applied to two sludge samples (herein referred to as US and SWE). The results for all fluorine analyses (TF, free IF, EOF, and target PFAS) on the US and SWE sludge samples are provided in Table 1. The TF concentration determined by the CIC was 4942 (RSD 2.7%) and 2231 (RSD 2.3%) μg/g F d.w., and the free IF concentration determined by the IC was 523 and 214 (RSD 7.6%) μg/g F d.w. for the US and SWE sludge, respectively. The EOF concentrations of the three different sample treatments (IPE, SPE, and Hx:Ac) ranged between 0.26 and 4.2 μg/g F d.w. The IPE method gave the highest concentrations of EOF for both the US and SWE sludges at 4.2 and 4.1 μg/g F d.w., followed by the SPE method with EOF concentrations of 3.3 and 2.1 μg/g F d.w., respectively. The Hx:Ac method gave approximately 10 times lower EOF concentrations (0.46 and 0.25 μg/g F d.w.) compared to the other two sample treatments (IPE and SPE). A similar trend was also observed for the target analysis of PFAS, where the IPE method gave the highest concentrations of PFAS (0.68 and 1.5 μg/g F d.w.), followed by SPE (0.44 and 0.64 μg/g F d.w.) and Hx:Ac (0.22 and 0.23 μg/g F d.w.). The lower PFAS concentrations in Hx:Ac compared to the other two methods might be because this method is more suitable for nonpolar organofluorine, such as polymeric PFAS. In this study, 85 PFASs were targeted, of which only two are stated as copolymers. Spaan et al.^36^ reported TF and EOF data on commercially available standard reference materials (SRMs), including the SRM NIST 2781, which is referred to in this study as the US sludge sample. The sample treatment used in that study involved a polar solvent (MeOH) liquid–solid extraction with an ENVI-carb cleanup, a method commonly used for PFAS analysis, to capture organofluorine of the anionic and neutral PFAS. The EOF concentration for the SRM NIST 2781 was 3.59 μg/g F d.w., aligning with present EOF concentrations for IPE and SPE for the SRM NIST 2781 (US sludge; Table 1). Contrary to the EOF results, the TF concentration in the present study was approximately six times higher compared to Spaan et al.^36^ Plausible explanations include differences in combustion conditions, calibration procedures, sample heterogeneity, and matrix-related interferences such as organic content and cations. While TF analysis obtains all fluorine species in the analysis, both inorganic and organic species, it may also increase the potential for matrix-related interferences. Cations, such as calcium, magnesium, potassium, and sodium, can form inorganic fluorine complexes such as fluorite (CaF_2_). These complexes have relatively high melting points (>1000 °C) and low solubility, which may influence HF formation and thereby the total fluorine levels. Furthermore, combustion efficiency for different fluorinated compounds might differ and influence the results. Total fluorine determination using CIC remains analytically challenging, particularly for complex environmental matrices such as sewage sludge. To our knowledge, no certified reference material or consensus TF value is currently available for fluorine mass balance analysis in sludge, and Spaan et al.^36^ is the only study besides this one to characterize total fluorine. Therefore, the present TF results should primarily be interpreted in a comparative context within this study rather than as absolute reference concentrations. Further investigation is necessary to better understand matrix-related interferences and factors such as inorganic compositions that may influence TF recovery in complex environmental samples.

**Table 1:**
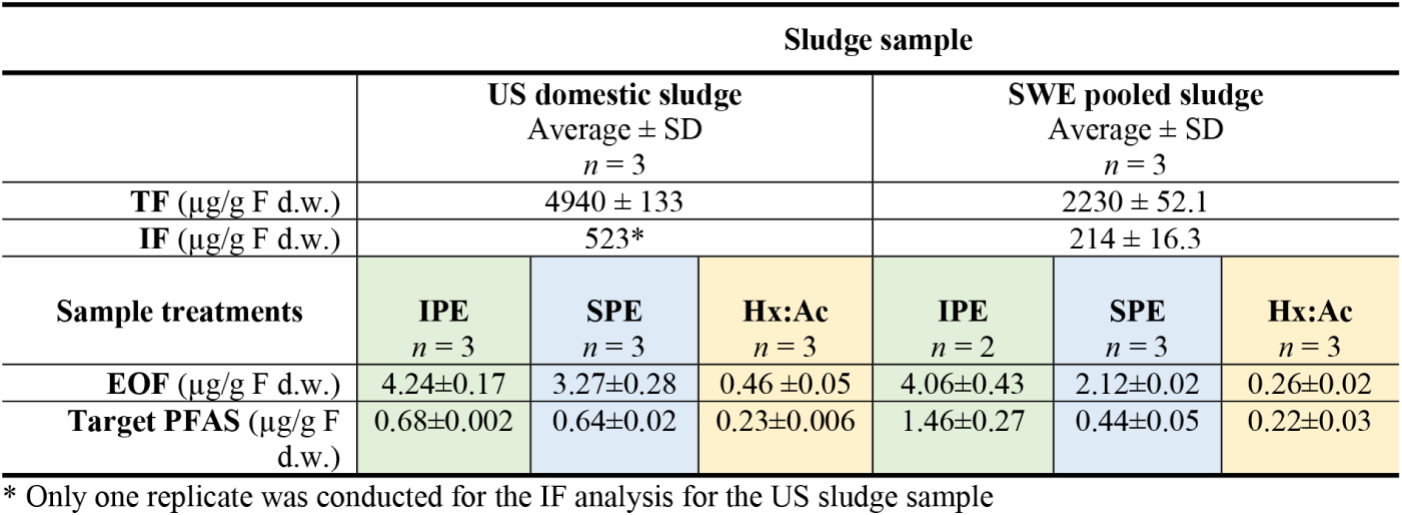
Concentrations (μg/g F d.w.) of Total Fluorine (TF), Free Inorganic Fluoride (IF), Extractable Organofluorine (EOF), and Target PFAS for the Three Sample Treatments (IPE, SPE, and Hx:Ac) in Two Sludge Samples (US Domestic Sludge (NIST SRM 2781) and SWE Pooled Sludge)

The fluorine mass balance concluded that the free IF explained 11% of the TF in the US sludge, leaving 89% to consist of organofluorine and nondissolved IF (Figure 1). A similar proportion was also observed for the SWE sludge, where free IF accounted for approximately 10%. The three sample treatments (IPE, SPE, and Hx:Ac) revealed that the EOF, regardless of which sample treatment was used, only accounted for less than 0.1% of the fluorine, and a major proportion of the fluorine remained nonextractable, which might be of organic origin (Table 1, Figure 1). The nonextractable fluorine may consist of inorganic fluorine species, strongly sorbed organofluorine compounds, and other unknown fluorinated compounds not recovered by the extraction method used. This fraction may also contain fluorinated compounds not efficiently recovered by the extraction methods used, including cationic PFAS and ultrashort-chain PFAS. Other fluorinated classes might include fluorinated polymers, unknown transformation products, pesticides, and pharmaceuticals. Furthermore, the nonextractable fluorine may consist of inorganic fluorine species that were not included in the dissolved inorganic fluorine measurement or inorganic anions. A recent study observed a high contribution of tetrafluoroborate and hexafluorophosphate in the mass balance.^37^ Whether such contributions to the nonextractable fluorine fraction in the present study remain unknown.

**Figure 1:**
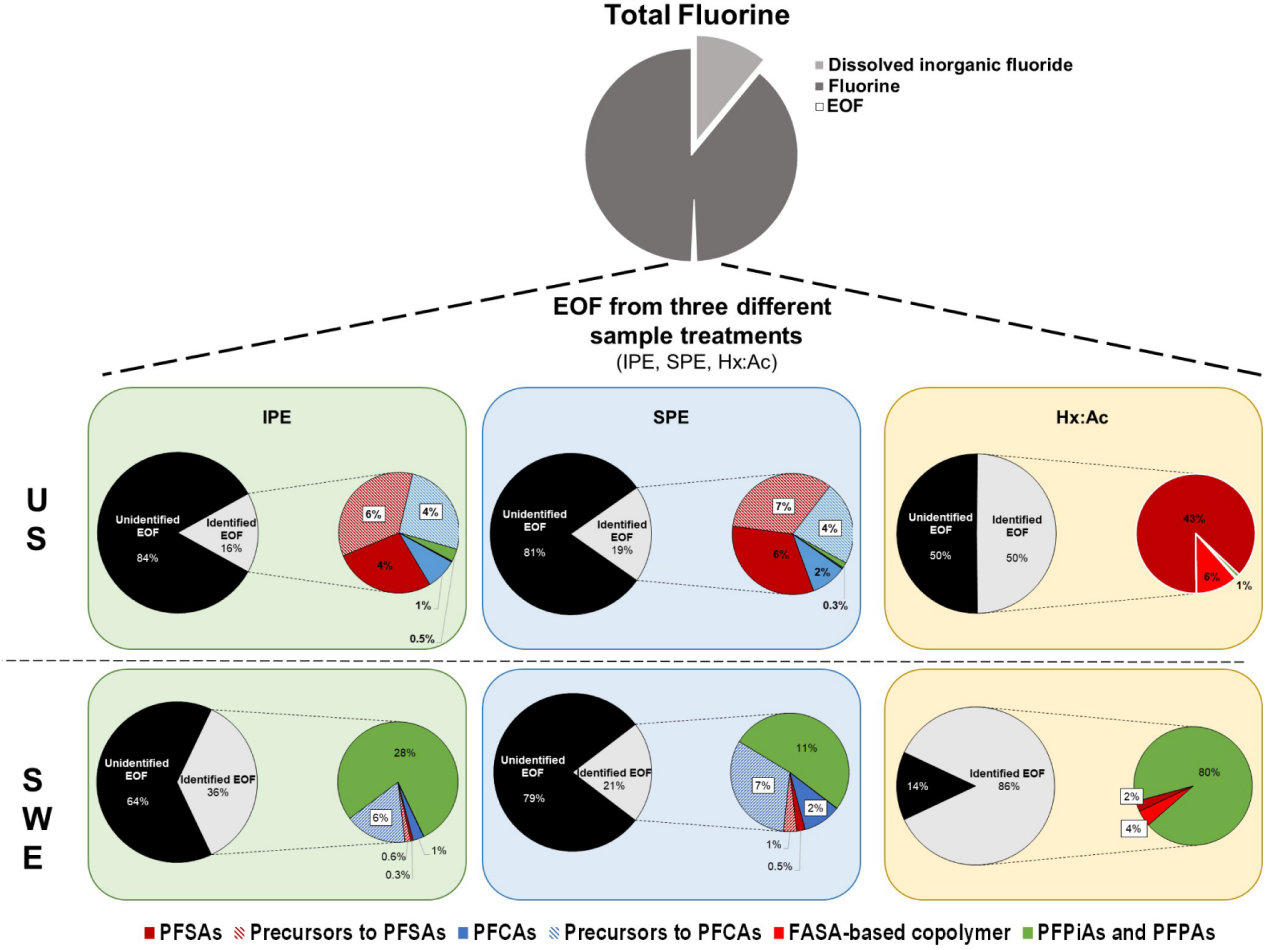
Fluorine mass balance determined by TF and IF in US sludge samples, together with its EOF mass balance for three different sample treatments (IPE, SPE, and Hx:Ac), was determined by EOF and target PFAS analysis. The lower part of the figure is the EOF mass balance for IPE, SPE, and Hx:Ac in the SWE sludge samples. All mass balance analyses are displayed as percentages (% after F equivalent conversion).

The concentrations of EOF and the composition of the identified part of EOF were observed to be highly dependent on the sample treatments (Figure 1). The IPE and SPE explained similar proportions of the EOF content, with similar profiles for the identified part in the US sludge. For the SWE sludge, the IPE method explains a higher proportion of the EOF content by target PFAS analysis compared to the SPE method (36% versus 21%). This was primarily due to the presence of PFPiAs and PFPAs, which were extracted with higher efficiency using the IPE method than with the SPE method. Similar observations regarding improved recovery of certain PFAS classes (i.e., PFPiAs and PFPAs) using ion-pair extraction compared to SPE-based approaches have been reported in a previous study.^34^ Another example illustrating the influence sample treatment has on EOF was that the FASA-based copolymers were only identified in the Hx:Ac method. The Hx:Ac was a sample treatment customized for these compounds and did not extract any PFCAs or their precursors. However, it also coextracted some of the PFSAs (extraction efficiency 80–95%, Table S4) and PFPiAs, making up a large proportion of the identified EOF in Hx:Ac (up to 86%). In conclusion, the present study illustrates EOF to be highly dependent on the sample treatment, aligning with a previous study evaluating EOF using two sample treatments on human blood.^34^ Furthermore, complementary sample treatments targeting different physicochemical properties may be necessary to capture the full range of EOF, fluorinated compounds, and PFAS chemistries.

### Extractable Organofluorine Mass in Swedish Sludge Samples

The IPE and Hx:Ac methods were selected for the Swedish sludge samples to capture a broader organofluorine content, including both polar and nonpolar organofluorine in sludge (as presented in the previous section). All 26 sludge samples showed detectable EOF concentrations with both methods. The EOF concentrations ranged between 535 and 14600 ng/g F d.w. for IPE and 40 and 950 ng/g F d.w. for Hx:Ac. The highest EOF concentration was for Borås in 2005 using the IPE method. All EOF concentrations were found to be at least five times higher with IPE compared to Hx:Ac, showing similar observations to the results for the US and SWE sludge samples (Table 1). The higher EOF concentrations for the IPE method may indicate a higher proportion of anionic organofluorine than nonpolar organofluorine in these sludge samples. Although relatively lower amounts of nonpolar organofluorine were observed, the Hx:Ac method might still obtain extractable polymeric organofluorine compounds with a low number of fluorine atoms relative to their high molecular mass. This fraction is often overlooked, and the knowledge about fluorinated polymers is limited, highlighting the importance of monitoring this fraction of organofluorines, potentially other copolymers (e.g., fluorotelomer-based acrylate polymers).^22^

The EOF mass balance profiles were conducted for all samples using both methods to estimate the proportion of unidentified organofluorines with different characteristics. All detectable PFAS concentrations included in the EOF mass balance can be found in the SI, Tables S6 and S7. The unidentified EOF was between 79 and 98% for the IPE method. Similar proportions of unidentified EOF were seen in Hx:Ac, with an average of 93% unidentified EOF. The main contributor to the identified portion was precursors to PFAS, specifically diPAPs for the IPE (15 to 56%), whereas for the Hx:Ac, up to 98% were identified as FASA-based copolymers. The high contribution of diPAPs to the identified EOF for the IPE method is consistent with an earlier study using SPE as a sample treatment on sewage sludge from Sweden, where diPAPs contributed up to 63% of the identified EOF.^38^

### Regional Comparison of Extractable Organofluorine Mass Balance in Swedish Sludge Samples Collected in 2015

A regional comparison of EOF levels was conducted by extracting four sludge samples collected in 2015 from four different WWTPs (Borås, Stockholm, Torsås, and Umeå) in Sweden (Figure 2). With the IPE method, Borås WWTP had the highest EOF concentration (2610 ng/g F d.w.), followed by Torsås (894 ng/g F d.w.), Stockholm (830 ng/g F d.w.), and Umeå (606 ng/g F d.w.). A similar pattern in EOF concentration was observed for the Hx:Ac method: Borås (212 ng/g F d.w.) > Torsås (109 ng/g F d.w.) > Umeå (102 ng/g F d.w.) > Stockholm (46 ng/g F d.w.). The higher EOF concentrations in Borås WWTP compared to the other three WWTPs for both IPE and Hx:Ac point to industrial activities in this area as a contributing factor. Since, Borås WWTP receives wastewater from several large textile industries.^39^ The present study results are contrary to a study reporting EOF concentrations in sludge samples from the same WWTPs collected in 2016.^38^ In that study, the EOF concentrations were observed in the following order: Stockholm (154 ng/g F d.w.) > Borås (56 ng/g F d.w.) > Umeå (<LOD). The differences in concentrations and ranking between studies may be related to differences in sample treatment, as the previous study only reported EOF of the anionic fraction using SPE. The IPE method relies on PFAS forming ion pairs with TBA and partitioning into the organic phase, whereas the SPE method retains PFAS on sorbent through electrostatic anion-exchange interaction. As a result of different extraction mechanisms, IPE may extract a broader range of fluorinated compounds, including more hydrophobic or nonconventional PFAS, while the SPE method generally provides cleaner and more selective extracts. For example, shorter-chain PFCAs are shown to have higher extraction efficiency with SPE compared to the IPE method, whereas the opposite can be seen for PFPiAs and PFPAs (Table S5). Figure 2 illustrates that a significant proportion of the two extraction methods remains unidentified, revealing that the unidentified EOF differs in characteristics (e.g., nonpolar and polar species).

**Figure 2:**
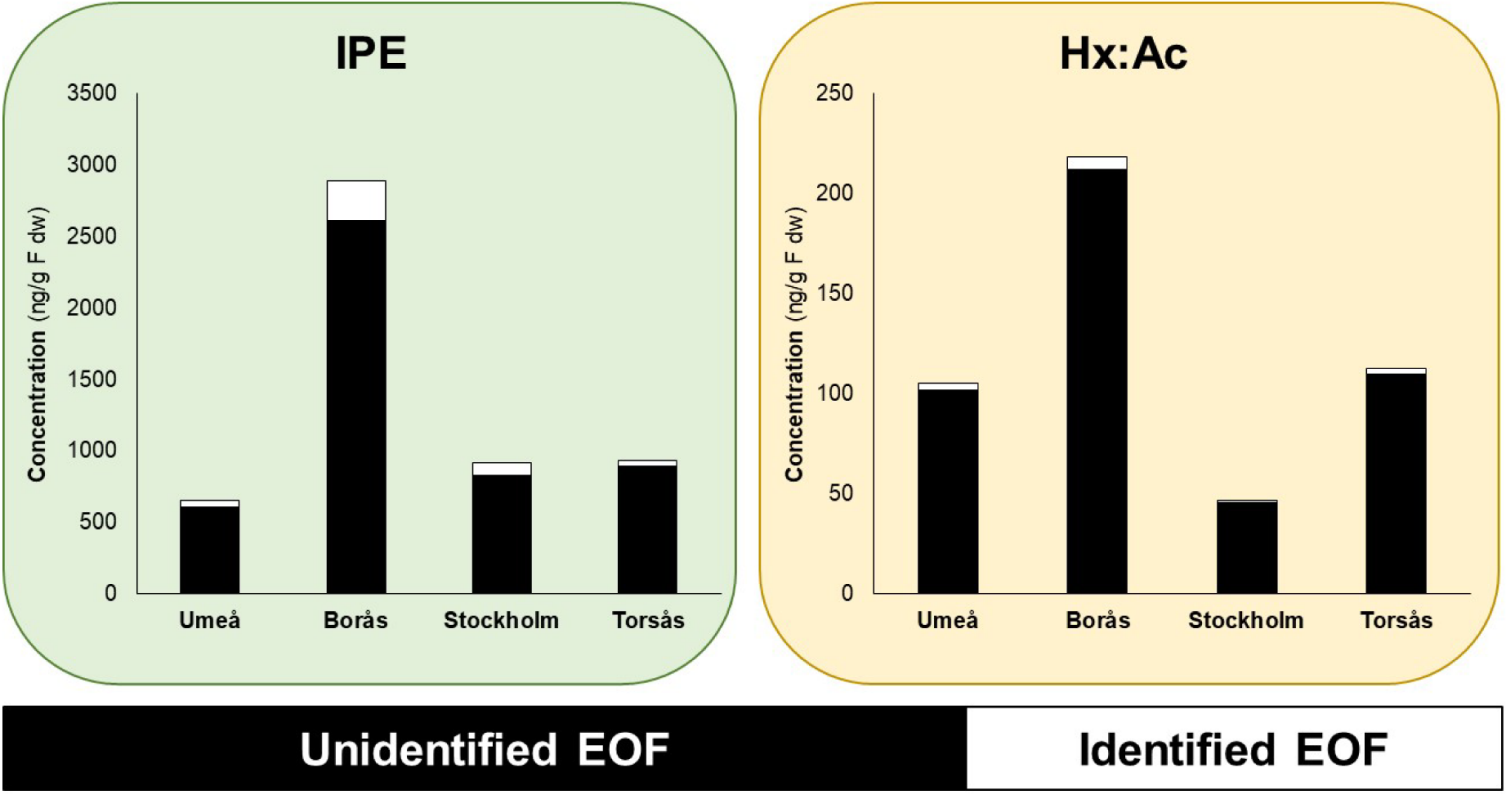
EOF mass balance determined by EOF and target analysis (ng/g F d.w.) in sludge samples from Umeå, Borås, Stockholm, and Torsås in 2015 for two different extraction methods (Hx:Ac method and IPE method).

### Temporal Analysis of EOF Mass Balance in Sludge

Temporal variation of EOF concentrations between 2004 and 2017 was studied in sludge samples from two of the four WWTPs in Sweden (Borås and Stockholm) using two different sample treatments (IPE and Hx:Ac). The EOF mass balance profiles, with the measured EOF concentrations for both extraction methods, can be seen for Stockholm in Figure 3 and for Borås in Figure 4. The concentrations of individual PFAS compounds can be found in SI, Table S6 and S7. Variations were observed in EOF concentrations between years, sites, and extraction methods (Figures 3 and 4). The EOF concentration in sludge from Stockholm was between 535 and 1270 ng/g F d.w. for the IPE method, whereas the Hx:Ac method gave an EOF range between 35 and 193 ng/g F d.w. For Borås, the EOF concentration varied between 1080 and 14600 ng/g F d.w. (IPE), and 125 and 952 ng/g F d.w. (Hx:Ac). All EOF concentrations were observed to be higher—up to 17 times—in sludge from Borås compared to sludge from Stockholm for the same year and sample treatment.

**Figure 3:**
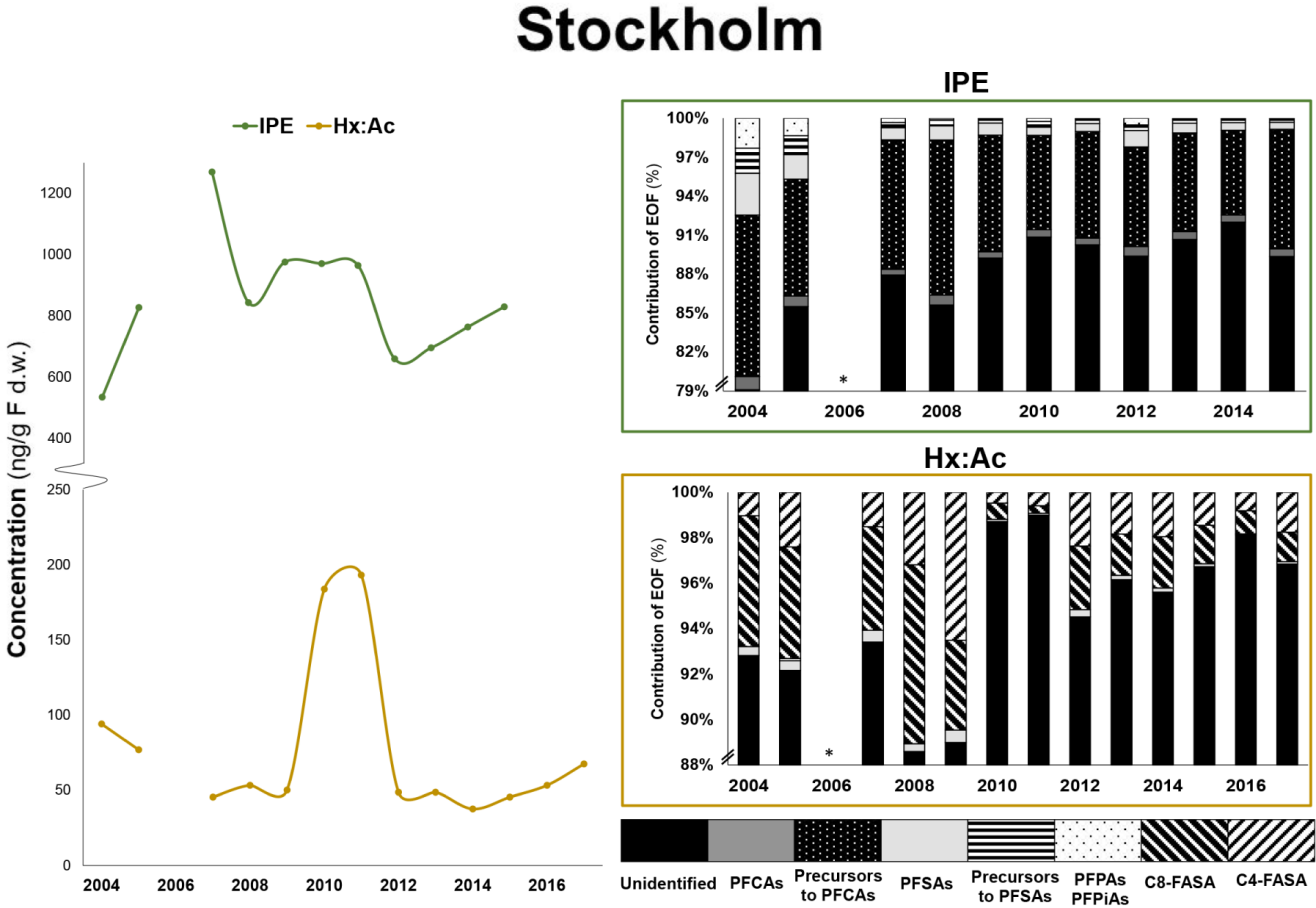
Temporal analysis of EOF mass balance (ng/g F d.w.) in sludge samples from Stockholm using two different extraction methods: IPE method (2004–2015) and the Hx:Ac method (2004–2017). The year 2006 is excluded and denoted with *. The right part of the figure is the composition of the identified EOF.

**Figure 4:**
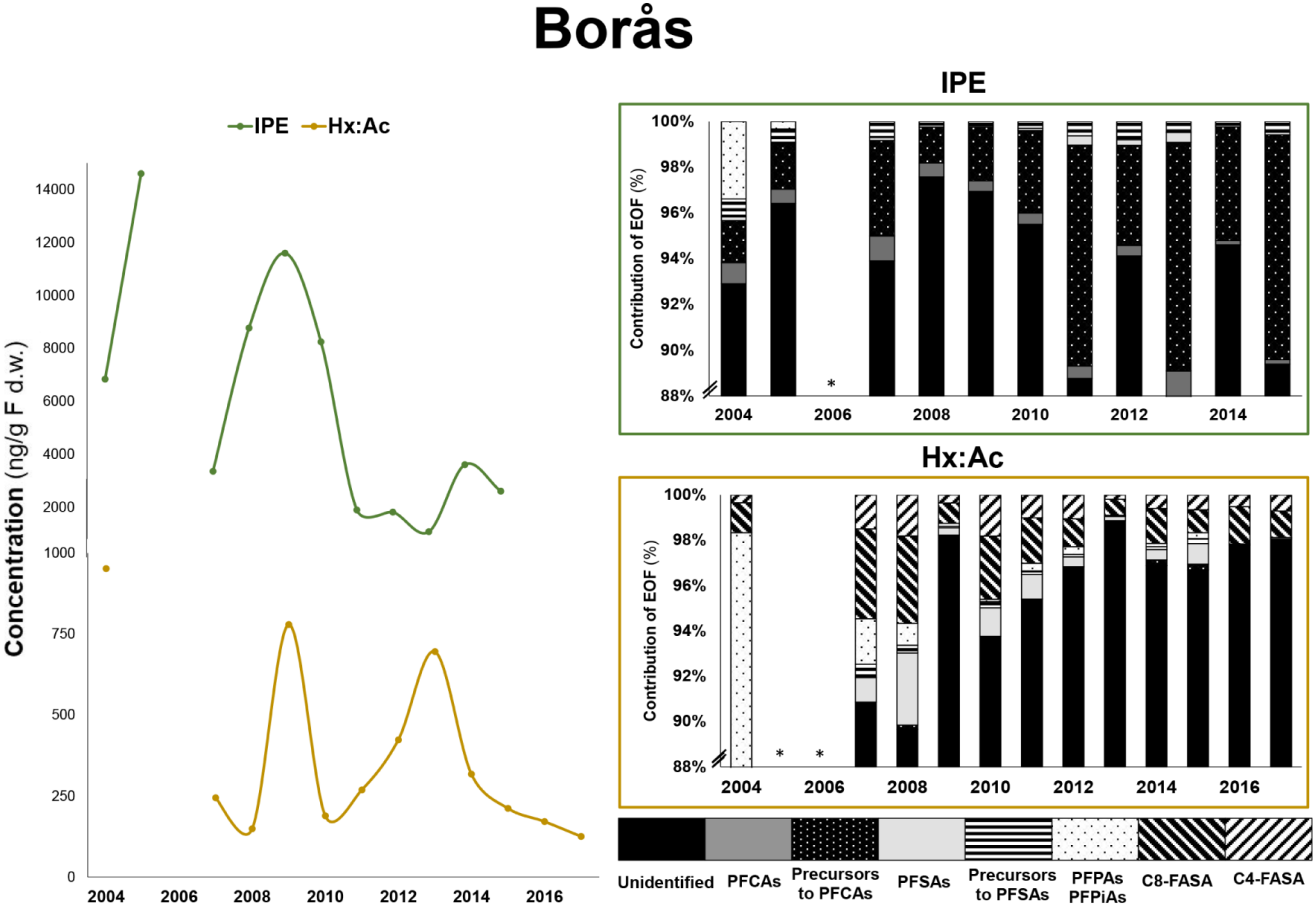
Temporal analysis of EOF mass balance determined by EOF and target analysis (ng/g F d.w.) in sludge samples from Borås using two different extraction methods: the Hx:Ac method (2004–2017) and the IPE method (2004–2015). The years excluded are denoted with *. The right part of the figure is the composition of the identified EOF.

For the IPE method, the target analysis of 83 anionic and neutral PFAS only accounted for a low proportion of the EOF. A majority of the EOF concentration in the sludge from Borås (87–98%) and Stockholm (79–92%) collected in 2004–2015 was unidentified. The more commonly monitored PFAS classes, such as PFCAs and PFSAs, contributed to an average of 0.8% of the EOF in Borås and 1.8% in Stockholm. Notably, the majority of the identified PFAS in the sludge from both WWTPs were attributed to nonconventional PFAS, more specifically PFCA and PFSA precursor compounds, intermediates, and PFPAs, which contributed on average 5.7% in Borås and 10% in Stockholm to the EOF. The contribution of nonconventional PFAS aligns with earlier studies.^38,40^ The low contribution of target PFAS to the total EOF was also observed for the Hx:Ac method customized for the FASA-based copolymers. The two FASA-based copolymers only contributed to a minor proportion (0.9–5.7%) of the EOF for Borås (Figure 4), whereas Stockholm had a higher contribution (up to 11%, Figure 3). The unidentified EOF ranged between 89% and 99% for Hx:Ac, except in 2004 at Borås (35%). In that year, relatively large amounts of PFPiAs were detected, which can explain the exception. In contrast, in the IPE method, high concentrations of PFPAs were found in the samples from 2004, but the PFPiAs were much lower. Stockholm had a higher contribution of the known PFAS to the total EOF for both extraction methods.

The EOF results from both extraction methods show that significant amounts of EOF remain unknown. Similar observations have been reported in other studies on different kinds of matrices.^30,33,40^ Overall, a declining trend of EOF concentrations for Borås WWTP was observed, whereas Stockholm WWTP showed steadier EOF concentrations. Nonetheless, results from both sample treatments indicate reduced concentrations of extractable organofluorine in sludge in recent years, which could be a result of the shift to more water-soluble PFAS (e.g., C6- and C4-chemistry). Previously, Yeung and Mabury^41^ reported an increasing trend of unidentified EOF in human blood, indicating that people are exposed to new and unknown fluorinated products in recent years. A similar trend regarding the unidentified EOF in the present study for both IPE and Hx:Ac was seen in sludge for Stockholm, going from a contribution of identified EOF of 21% and 7% in 2004 to 11% and 3% in 2015, respectively. The increasing proportion of unidentified EOF between 2004 and 2017 might reflect a shift in the composition of fluorinated compounds entering the WWTPs over time. During this period, regulatory actions and voluntary phase-outs targeting legacy long-chain PFAS,^42,44^ likely contributed to a reduction in the proportion of known EOF. However, these compounds have likely been replaced by other fluorinated compounds, including shorter-chain, fluorinated polymers and other fluorinated compounds not included in the target PFAS analysis, resulting in a higher proportion of unidentified EOF in later years. Similar observations have been reported in previous studies, where target PFAS accounted for only a limited fraction of EOF.^41–45^ These observations suggest that target analysis alone may underestimate the diversity and extent of extractable fluorinated compounds, particularly as PFAS use patterns shift toward alternative and less well-characterized fluorinated substances. Further studies are needed to clarify this point and to characterize the unknown fluorinated proportion and its potential sources.

To conclude, all sludge samples in the present study contained significant amounts of nonpolar, polar, and anionic organofluorines. The low proportion of all identified PFAS observed in this study shows a similar pattern to earlier WWTP studies,^38,46–48^ which indicates that a high amount of other organofluorines is ignored and potentially constitutes an environmental risk.

### Characteristics of the Unidentified Organofluorine—Limitations and Implications

The present EOF results further illustrate that EOF is highly dependent on the sample treatment used; therefore, when comparing EOF results between different studies, sample treatment must be taken into consideration. In the present study, the EOF concentrations were up to 1.5 to 43 times higher in the IPE method compared with the Hx:Ac method when comparing sludge samples from the same site and year. The higher concentration of EOF in IPE may indicate the presence of a higher proportion of anionic organofluorine than nonpolar organofluorine in these sludge samples. This study demonstrates a new comprehensive workflow using two extraction methods, which, in turn, give complementary guidance into the unknown organofluorine characteristics. Both extraction methods contained only a minor proportion that could be explained by the target analysis, demonstrating that the unidentified EOF is likely to be organofluorines with different properties (both polar and nonpolar organofluorine).

Even though different sample treatments result in different contributions of PFAS classes and EOF, a large proportion remains unidentified EOF. Contributors to the EOF content in the sludge, other than anionic and neutral PFAS, may be fluorinated agrochemicals or pharmaceuticals. It has been estimated that globally, about 30% of pharmaceuticals and pesticides contain organofluorine, and the production volume of intermediates for pharmaceuticals and pesticides was up to 60000 tons in 2003.^49^ It is not clear whether current extraction methods might have captured these compounds. To further investigate this, two fluorinated pharmaceuticals (fluoxetine and celecoxib) were analyzed in the SWE sludge sample. Interestingly, both fluoxetine and celecoxib were found with the IPE method, whereas only celecoxib was observed for Hx:Ac (Table S5). A study by Spaan et al.^50^ increased their contribution of identified EOF to 27% from 2% by including numerous fluorinated pharmaceuticals and pesticides. In the present study, lower contributions were observed in the pooled Swedish sludge (<1% of the total EOF). However, in the current investigation, only two fluorinated pharmaceuticals were targeted, and their metabolites were not measured either.

Other organofluorine contributors may be fluorinated polymers or their monomers. In 2018, over 320000 tons of fluoropolymers were produced by industries,^51^ and the production of fluoropolymers was observed to increase rapidly by 5 to 8% per year.^52^ In the present study, only two FASA-based copolymers were analyzed for and found in all sludge samples treated with the Hx:Ac method. In Sweden, fluorinated polymers represent the largest share of over 3000 identified commercial PFASs.^53^ Whether these fluorinated polymers were extracted with the present study methods is still unknown and needs to be further studied. The EOF concentrations reported from the two methods give a more comprehensive representation of the possible PFAS contamination. However, this may still not represent the whole picture.

The EOF mass balance analysis is a crucial screening approach to give a broader picture of the occurrence of PFASs and other organofluorines. Only measuring selective PFAS compounds does not show a representative exposure to the overall usage of PFAS and, therefore, results in the risk of underestimating their effects on humans and the environment. There is a considerable knowledge gap in the characterization and identification of the unknown EOF, and further studies are needed to understand the associated risks. Multiplatform approaches, such as a combination of nontarget analysis using a suspect screening workflow together with oxidative conversion, will give better knowledge about the overlooked PFAS.

## Supplementary Material



## Notes

Information on chemicals, sample treatments, instrumental analysis, quality assurance and quality control, results from the influence of sample treatment on EOF, and sludge sample concentrations (PDF)
