# Supplementary material for: Spatial and Temporal Analysis of Fluorine Mass Balance in Swedish Sludge and the Impact of Sample Treatment on Extractable Organofluorine
Source: Environ Sci Technol. 2026 Jul 21;60(30):21247–56. doi: 10.1021/acs.est.6c03578 (PMC13449884; doi:10.1021/acs.est.6c03578)
Supplement: Supplementary file 1 [file es6c03578_si_001.pdf]

Supporting Information

Spatial and temporal analysis on fluorine mass balance in Swedish sludge and impact of sample treatment on extractable organofluorine

Felicia Fredriksson, Ulrika Eriksson, Anna Kärrman, Leo WY Yeung*

Man-Technology-Environment (MTM) Research Centre, School of Science and Technology, Örebro University, Sweden, SE-701 82

* Corresponding author: leo.yeung@oru.se

Document Summary: 24 pages, 7 SI tables (Tables S1 – S7)

Table of Contents

Materials and Methods 3

Chemicals and Reagents 3

Sample Extraction and Clean-up 4

Instrumental Analysis 7

Quality Assurance and Quality Control 14

Influence of sample treatment on EOF 15

Target PFAS levels used to determine how different sample treatments influence EOF 17

Target PFAS levels in sludge used for the fluorine mass balance analysis 20

Materials and Methods

Chemicals and Reagents

A list of target analytes including native perfluoroalkane sulfonates (PFSAs: C3-10, 12), mass-labelled PFSAs (C4, C6, C8), native perfluorinated carboxylates (PFCAs: C2-C14, C16, C18), mass-labelled PFCAs (C4-C12, C14, C16), native fluorotelomer sulfonates (FTSAs: 4:2, 6:2, 8:2), mass-labelled FTSAs (6:2, 8:2), native fluorotelomer unsaturated carboxylates (FTUCAs: 6:2, 8:2, 10:2), mass-labelled FTUCAs (6:2, 8:2, 10:2), native perfluorooctanesulfonamidoacetic acids (FOSAAs) (FOSAA, MeFOSAA, EtFOSAA), mass-labelled EtFOSAA, native polyfluoroalkyl phosphate monoesters/diesters (6:2 PAP, 8:2 PAP, 6:2 diPAP, 8:2 diPAP, 6:2/8:2 diPAP, 10:2 monoPAP, 10:2 diPAP), mass-labelled PAPs (6:2 monoPAP, 8:2 monoPAP, 6:2 diPAP, 8:2 diPAP), diethyl perfluorooctanesulfonamidoethanol (EtFOSE)-based polyfluoroalkyl phosphate ester (diSAmPAP) and ethyl perfluorooctanesulfonamidoethanol (EtFOSE)-based polyfluoroalkyl phosphate ester (SAmPAP), native perfluorinated phosphonates (PFPAs: C6, C8, C10), native perfluorinated phosphinates (PFPiAs: 6:6, 6:8, 8:8) were obtained from Wellington Laboratories (Guelph, Ontario, Canada). Other analytical standards were purchased from other companies; 10:2 mono- and di-PAP from Chiron (Trondheim, Norway); trifluoromethane sulfonic acid (TFMS) was from Sigma-Aldrich (Stockholm, Sweden), perfluoroethane sulfonic acid (PFEtS) in form of potassium salt was purchased from Kanto Chemicals Xo., Inc., (Portland, USA), trifluoroacetic acid (TFA) was from Sigma-Aldrich (Munich, Germany), and perfluoropropanoic acid (PFPrA) was purchased from Sigma-Aldrich (Oakville, Canada). Standards used for analysis of the fluoroalkyl sulfonamide (FASA)-based copolymers were technical mixtures of Scotchgard™ pre-2002 formulation (100 µg/mL in methanol) and Scotchgard™ post-2002 formulation (100 µg/mL in methanol) purchased from AccuStandard Inc. (New Haven, USA). These two technical mixtures were prepared from commercial

products of Scotchgard™ fabric protector produced before 2002 and after 2002 by the 3M Company. The two fluorinated pharmaceuticals (fluoxetine, celecoxib) were purchased from Sigma-Aldrich (St. Louis, USA). Mass-labeled TFA was obtained from Toronto Research Chemicals Inc., (Toronto, Canada).

HPLC-grade methyl *tert*-butyl ether (MTBE, ≥99 %), tetrabutylammonium bisulfate (TBA) and ammonium acetate were purchased from Sigma Aldrich; sodium hydroxide (≥99 %), analytical grade ammonium hydroxide (25%), HPLC-grade acetonitrile (can, (≥99.9 %), isopropanol and HPLC- and LCMS-grade methanol (MeOH, ≥99.8 % and ≥99.9 %) were from Fisher Scientific (Ottawa, Canada); hydrochloric acid was from Scharlau Chemie S.A (Spain); 1-methylpiperidine was purchased from Sigma Aldrich (Stockholm, Sweden). Scientific HPLC-grade acetone (>98.9%) and dichloromethane (DCM, ≥99.8 %) from Saveen-Werner (Germany), *n*-hexane (≥ 99.0 %) from VWR International (Darmstadt, Germany) and laboratory-produced ultrapure water (18.2 MΩ) were used. Disposable silica gel solid-phase extraction (SPE) columns (500mg, 3 mL; J.T. Baker®) purchased from GTF/Fisher Scientific (Gliwice, Poland), Supel™ QuE Z-Sep sorbent obtained from Sigma-Aldrich (Bellefonte, USA), and Oasis WAX cartridges (150 mg, 6 mL, 30 µm) and GHP filters (0.2 µm) purchased from Waters Corporation (Milford, USA) were used.

Sample Extraction and Clean-up

Alkaline Digestion Extraction – ion pair or solid phase cleanup

The freeze-dried sludge was weighed (0.25 ± 0.005 g) and extracted. The extraction was performed by adding 2 mL 1M sodium hydroxide in methanol and the samples were vortexed,

ultrasonicated for 15 minutes, shaken for 15 minutes at 250 rpm, and then centrifuged at 8000 g. The supernatant was transferred to a new 15 mL polypropylene tube. The extraction was repeated twice with 2 mL methanol, and all extraction aliquots were combined, and gently evaporated to 0.5 mL under nitrogen gas, before clean-up. The samples were purified with either ion pair extraction (IPE) or solid phase extraction (SPE).

Ion pair extraction. The cleanup started by adding 2 mL of 0.5M TBA solution into the extract. The mixture was vortexed before 3 mL of MTBE was added, then vortex mixed again and shaken for 15 minutes at 250 rpm, and then centrifuged at 8000 g for 10 min. The organic phase was transferred to a new 15 mL polypropylene tube, and the clean-up procedure was repeated twice. The combined MTBE was evaporated gently under nitrogen gas to dryness and reconstituted in 0.5 mL methanol. The final extract was split for LC-MS/MS and CIC analysis.

Solid phase extraction. The 0.5 mL extract was first diluted with 10 mL ultrapure water (18.2 MΩ) before cleanup by SPE using Oasis Weak Anion Exchange (WAX) cartridges (Waters Corporation, Milford, USA). The sorbent was conditioned with 4mL 0.1% NH_4OH in methanol, followed by 4 mL methanol, and 4 mL ultrapure water. The diluted extract was loaded on the sorbents and thereafter washed with 20 mL 0.01% NH_4OH in ultrapure water, 30 mL ultrapure water, and 4 mL ammonium acetate buffer solution (pH 4) in a series. The cartridges were dried under a vacuum for 30 minutes. The analytes were eluted with 2 mL methanol followed by 4 mL 0.1% NH_4OH in methanol. The combined eluents were evaporated gently under nitrogen gas to 0.5 mL and the final extract was split for LC-MS/MS and CIC analysis.

Hexane:Acetone Extraction

The freeze-dried sludge was weighed (0.25 ± 0.005 g) and extracted. The extraction was performed three times using 8 mL of acetone:hexane (1:1, v/v), vortexed, ultrasonicated for 15 minutes, and then centrifuged at 6000 g for 15 minutes. All extraction aliquots were combined, gently evaporated to dryness under nitrogen gas, and reconstituted with 2 mL of DCM. The extract was purified using a disposable silica gel SPE cartridge (500 mg, J.T. Baker®).

The silica gel SPE cartridges were preconditioned by 3 mL DCM. The sample extract was loaded on the cartridge, followed by washing the sorbent with 6 mL DCM. After washing, 2 mL of AcN:DCM (40:60, v/v) was added to each cartridge to elute the target analytes. The eluent was collected, evaporated gently to dryness under nitrogen gas, and reconstituted with 2 mL of AcN. The reconstituted extract was vortex mixed and ultrasonicated for 20 minutes.

Dispersive- SPE was used for further purification by adding approximately 200 mg of Supel QuE Z-Sep sorbent to the reconstituted extract that was vortex mixed for 60 seconds. The extract with the sorbent was gently evaporated to around 1 mL under nitrogen gas and then filtered with a 0.2 µm GHP filter before it was further evaporated to dryness under nitrogen gas and reconstituted with 0.5 mL of MeOH. The extract was split for LC-MS/MS and CIC analysis.

Instrumental Analysis

The instrumental analysis was performed on an Acquity Ultra Performance Liquid Chromatograph (UPLC) system coupled to a XEVO TQ-S tandem mass spectrometer (MS/MS; Water Corporation, Milford, USA), in negative electrospray ionization. Gradient elution was applied on a 100 mm C18 BEH column (1.7 µm, 2.1 mm; Water Corporation, Milford, USA). The mobile phases consisted of 2 mM ammonium acetate in Milli-Q water: MeOH (70:30) (A) and MeOH (B). For diPAPs analysis addition of 5 mM 1-methyl piperidine as an additive was used. All analytes were analyzed using multiple reaction monitoring (MRM), with at least two transitions, except for perfluorobutanoic acid (PFBA) and perfluoropentanoic acid (PFPeA) where one transition was monitored. Qualification of fluorinated pharmaceuticals (fluoxetine, celecoxib), and ultrashort chain PFAS (C2-C3 PFCAs and C1-C3 PFSAs) was done by an Acquity Ultra Performance Convergence Chromatograph (UPC2) system coupled to Xevo TQ-Sµ tandem mass spectrometer (Water Corporation, Milford, USA).

Table S 1. Mass spectrometric settings used for target PFAS analysis grouped in their compound classes; including MRM transitions, cone voltage, collision energy, and its corresponding internal standard used

Analyte	*Precursor/ product ions quantification (m/z)*	*Cone (V)*	*Col (eV)*	*Precursor/ product ions qualification (m/z)*	*Cone (V)*	*Col (eV)*	*Corresponding Internal Standard*
**Precursors to perfluoroalkyl sulfonic acids**							
FBSA	297.90/77.92	20	20	297.90/118.94	20	15	$^{13}C_4$ PFBA
Me-FBSA	311.97/111.93	14	20	311.97/218.95	14	16	$^{13}C_4$ PFBA
FHxSA	397.90/77.92	30	26	397.90/168.94	30	28	$^{18}O_2$ PFHxS
Me-FHxSA	411.97/168.93	24	24	411.97/318.96	24	20	$^{18}O_2$ PFHxS
FOSA	497.90/78.00	82	30	497.90/168.96	82	28	$^{13}C_8$ FOSA
MeFOSA	512/169	27	45	-	-	-	$^{2}H_3$ MeFOSA
EtFOSA	526/169	27	45	-	-	-	$^{2}H_3$ MeFOSA
MeFOSE	616/59	27	45	556.03/121.99	42	34	$^{2}H_7$ MeFOSE
EtFOSE	630/59	27	45	570.1/135.98	48	32	$^{2}H_7$ MeFOSE
FOSAA	555.84/482.76	60	26	555.84/418.85<br>555.84/218.90	60	24<br>30	$^{2}H_5$ EtFOSAA
MeFOSAA	569.78/418.87	16	18	569.78/482.76<br>569.78/511.92	16	14<br>22	$^{2}H_5$ EtFOSAA
EtFOSAA	583.84/418.84	18	20	583.84/482.80<br>583.84/525.88	18	16<br>20	$^{2}H_5$ EtFOSAA
SAmPAP	649.78/96.87	2	26	649.78/168.91	2	36	$^{13}C_2$ 8:2monoPAP
diSAmPAP	1202.65/525.87	92	46	1202.65/168.90<br>1202.65/218.90	92	64<br>66	$^{13}C_4$ 8:2 diPAP
**Perfluoroalkyl sulfonic acids** (PFSAs)							
PFBS	298.90/98.90	20	26	298.90/79.96	20	26	$^{13}C_3$ PFBS
PFPeS	348.90/98.96	20	26	348.90/79.96	20	30	$^{18}O_2$ PFHxS
PFHxS	398.90/98.90	20	30	398.90/79.96	20	34	$^{18}O_2$ PFHxS
PFHpS	448.97/98.90	20	30	448.97/79.96	20	35	$^{13}C_4$ PFOS

Analyte	***Precursor/ product ions quantification*** *(m/z)*	***Cone (V)***	***Col (eV)***	***Precursor/ product ions qualification*** *(m/z)*	***Cone (V)***	***Col (eV)***	***Corresponding Internal Standard***
**Perfluoroalkyl sulfonic acids** (PFSAs)							
PFOS	498.97/98.96	20	38	498.97/79.96<br>498.97/169.03	20	44<br>34	$^{13}C_4$ PFOS
PFNS	548.90/98.96	20	38	548.90/79.96	20	44	$^{13}C_4$ PFOS
PFDS	598.97/98.90	20	42	598.97/79.96	20	58	$^{13}C_4$ PFOS
PFDoDS	698.97/98.90	20	40	698.97/79.96	20	45	$^{13}C_4$ PFOS
**Precursors to perfluoroalkyl carboxylic acids**							
4:2 FTSA	327.00/307.00	20	20	327.00/81.00	20	28	$^{13}C_2$ 6:2 FTSA
6:2 FTSA	427.00/407.00	20	20	427.00/81.00	20	28	$^{13}C_2$ 6:2 FTSA
8:2 FTSA	527.00/507.00	20	20	527.00/80.00	20	28	$^{13}C_2$ 8:2 FTSA
10:2 FTSA	627.00/607.00	20	20	627.00/81.00	20	28	$^{13}C_2$ 8:2 FTSA
3:3 FTCA	240.90/116.98	10	30	240.90/176.98<br>240.90/59.07	10	8<br>14	$^{13}C_2$ 6:2 FTUCA
5:3 FTCA	340.90/236.97	10	16	340.90/216.93	10	22	$^{13}C_2$ 6:2 FTUCA
7:3 FTCA	440.90/336.89	12	14	440.90/316.93	12	20	$^{13}C_2$ 8:2 FTUCA
6:2 FTUCA	356.90/292.91	10	18	356.90/242.95	10	36	$^{13}C_2$ 6:2 FTUCA
8:2 FTUCA	456.90/392.94	10	18	456.90/342.88	10	38	$^{13}C_2$ 8:2 FTUCA
10:2 FTUCA	556.84/492.82	8	16	556.84/242.94	8	38	$^{13}C_2$ 10:2 FTUCA
6:2 monoPAP	442.90/96.95	10	18	442.90/422.89	10	12	$^{13}C_2$ 6:2monoPAP
8:2 monoPAP	542.90/97	22	14	542.90/522.90	22	14	$^{13}C_2$ 8:2monoPAP
10:2 monoPAP	642.97/97.00	24	28	642.97/525.83	24	22	$^{13}C_2$ 8:2monoPAP
4:2 diPAP	588.90/97.00	64	28	588.90/342.91	64	18	$^{13}C_4$ 6:2 diPAP
4:2/6:2 diPAPs	688.90/97.00	64	28	688.90/342.91<br>688.90/442.91	64	18	$^{13}C_4$ 6:2 diPAP
2:2/8:2 diPAPs	688.90/97.00	64	28	688.90/242.91<br>688.90/542.91	64	18	$^{13}C_4$ 6:2 diPAP
6:2 diPAP	788.90/97.00	64	28	788.90/442.91	64	18	$^{13}C_4$ 6:2 diPAP

Analyte	***Precursor/ product ions quantification*** *(m/z)*	***Cone (V)***	***Col (eV)***	***Precursor/ product ions qualification*** *(m/z)*	***Cone (V)***	***Col (eV)***	***Corresponding Internal Standard***
**Precursors to perfluoroalkyl carboxylic acids**							
4:2/8:2 diPAP	788.90/97.00	64	28	788.90/342.91<br>788.90/542.91	64	18	$^{13}C_4$ 6:2 diPAP
2:2/10:2 diPAP	788.90/97.00	64	28	788.90/242.91<br>788.90/642.91	64	18	$^{13}C_4$ 6:2 diPAP
8:2 diPAP	988.78/96.94	68	34	988.78/542.81	68	26	$^{13}C_4$ 8:2 diPAP
6:2/10:2 diPAP	988.78/96.94	68	34	988.78/442.81<br>988.78/642.81	68	26	$^{13}C_4$ 8:2 diPAP
4:2/12:2 diPAP	988.78/96.94	68	34	988.78/342.81<br>988.78/742.81	68	26	$^{13}C_4$ 8:2 diPAP
6:2/8:2 diPAP	888.78/96.94	66	34	888.78/442.81<br>888.78/542.81	66	26	$^{13}C_4$ 6:2 diPAP
4:2/10:2 diPAP	888.78/96.94	66	34	888.78/342.81<br>888.78/642.81	66	26	$^{13}C_4$ 8:2 diPAP
8:2/10:2 diPAP	1088.78/96.94	68	34	1088.78/542.81<br>1088.78/642.81	68	26	$^{13}C_4$ 8:2 diPAP
6:2/12:2 diPAP	1088.78/96.94	68	34	1088.78/442.81<br>1088.78/742.81	68	34	$^{13}C_4$ 8:2 diPAP
10:2 diPAP	1188.78/96.94	68	34	1188.78/642.81	68	26	$^{13}C_4$ 8:2 diPAP
8:2/12:2 diPAP	1188.78/96.94	68	34	1188.78/742.81<br>1188.78/542.81	68	26	$^{13}C_4$ 8:2 diPAP
6:2/14:2 diPAP	1188.78/96.94	68	34	1188.78/842.81<br>1188.78/442.81	68	26	$^{13}C_4$ 8:2 diPAP
10:2/12:2diPAP	1288.78/96.94	68	34	1288.78/642.81<br>1288.78/742.81	68	26	$^{13}C_4$ 8:2 diPAP
8:2/14:2 diPAP	1288.78/96.94	68	34	1288.78/542.81<br>1288.78/842.81	68	26	$^{13}C_4$ 8:2 diPAP
12:2 diPAP	1388.78/96.94	68	34	1388.78/742.81	68	26	$^{13}C_4$ 8:2 diPAP
10:2/14:2diPAP	1388.78/96.94	68	34	1388.78/642.81<br>1388.78/842.81	68	26	$^{13}C_4$ 8:2 diPAP
8:2/16:2 diPAP	1388.78/96.94	68	34	1388.78/542.81<br>1388.78/942.81	68	26	$^{13}C_4$ 8:2 diPAP
**Perfluoroalkyl carboxylic acids** (PFCAs)							
PFBA	212.97/169.00	20	11	-	-	-	$^{13}C_4$ PFBA
PFPeA	262.97/219.00	20	8	-	-	-	$^{13}C_3$ PFPeA
PFHxA	312.97/269.00	20	9	312.97/118.95	20	26	$^{13}C_2$ PFHxA
PFHpA	362.97/319.00	20	10	362.97/168.97	20	16	$^{13}C_2$ PFHxA

Analyte	*Precursor/ product ions quantification (m/z)*	*Cone (V)*	*Col (eV)*	*Precursor/ product ions qualification (m/z)*	*Cone (V)*	*Col (eV)*	*Corresponding Internal Standard*
**Perfluoroalkyl carboxylic acids** (PFCAs)							
PFOA	412.97/369.00	20	10	412.97/168.97	20	18	$^{13}C_4$ PFOA
PFNA	462.99/419.00	20	12	462.99/219.00	20	18	$^{13}C_5$ PFNA
PFDA	512.97/469.00	20	11	512.97/219.00	20	18	$^{13}C_2$ PFDA
PFUnDA	562.97/519.00	20	12	562.97/268.99	20	18	$^{13}C_2$ PFUnDA
PFDoDA	612.97/569.00	34	14	612.97/168.96	40	22	$^{13}C_2$ PFDoDA
PFTrDA	662.90/619.00	20	14	662.90/168.96	20	26	$^{13}C_2$ PFDoDA
PFTDA	712.90/669.00	20	14	712.90/168.97	20	28	$^{13}C_2$ PFTDA
PFHxDA	812.90/769.00	30	15	812.90/168.96	42	32	$^{13}C_2$ PFHxDA
PFOcDA	912.90/869.00	36	15	912.90/168.96	36	36	$^{13}C_2$ PFHxDA
**Perfluoroalkyl phosphonic acids** (PFPAs)							
PFHxPA	398.97/79.00	62	26	-	-	-	$^{13}C_4$ PFOA
PFOPA	499.00/79.00	62	30	-	-	-	$^{13}C_4$ PFOA
PFDPA	599.03/79.00	62	34	-	-	-	$^{13}C_5$ PFNA
PFDoPA	699/79	62	30	-	-	-	^{13}C-PFOA
PFTePA	799/79	62	30	-	-	-	^{13}C-PFOA
PFHxDPA	899/79	62	30	-	-	-	^{13}C-PFOA
**Perfluoroalkyl phosphinic acids** (PFPiAs)							
6:6 PFPiA	701.00/401.00	24	28	-	-	-	$^{13}C_2$ PFDoDA
6:8 PFPiA	801.00/401.00	24	28	801.00/501.00	24	28	$^{13}C_2$ PFTDA
8:8 PFPiA	901.00/501.00	24	28	-	-	-	$^{13}C_2$ PFTDA
6:10 PFPiA	1001/401	24	28	1001/601	24	28	^{13}C-PFDoDA
8:10 PFPiA	1101/501	24	28	1101/601	24	28	^{13}C-PFDoDA
6:12 PFPiA	1101/401	24	28	1101/701	24	28	^{13}C-PFDoDA

Analyte	***Precursor/ product ions quantification*** *(m/z)*	***Cone (V)***	***Col*** *(eV)*	***Precursor/ product ions qualification*** *(m/z)*	***Cone (V)***	***Col*** *(eV)*	***Corresponding Internal Standard***
**Perfluoroalkyl phosphinic acids** (PFPiAs)							
10:10 PFPiA	1201/601	24	28	-	-	-	^{13}C-PFDoDA
8:12 PFPiA	1201/601	24	28	1201/701	24	28	^{13}C-PFDoDA
6:14 PFPiA	1201/401	24	28	1201/801	24	28	^{13}C-PFDoDA
10:12 PFPiA	1301/601	24	28	1301/701	24	28	^{13}C-PFDoDA
8:14 PFPiA	1301/501	24	28	1301/801	24	28	^{13}C-PFDoDA
12:12 PFPiA	1401/701	24	28	-	-	-	^{13}C-PFDoDA
10:14 PFPiA	1401/601	24	28	1401/801	24	28	^{13}C-PFDoDA
14:14 PFPiA	1501/701	24	28	-	-	-	^{13}C-PFDoDA
**Novel PFAS**							
8:2 Cl-PFESA	630.90/450.98	58	24	630.90/83.03<br>630.90/98.95	58	24<br>26	$^{13}C_4$ PFOS
6:2 Cl-PFESA	530.90/350.98	58	24	530.90/83.03<br>530.90/98.95	58	24<br>26	$^{13}C_4$ PFOS
T-PFECHS	460.84/380.94	2	24	460.84/98.88<br>460.84/118.96	2	26<br>38	$^{13}C_4$ PFOA
HFPO-DA	284.92/168.72	20	7	328.95/184.80	20	17	$^{13}C_3$ HFPO-DA
ADONA	376.97/250.80	30	37	376.97/84.69	15	29	$^{18}O_2$ PFHxS
**Side-chain fluorinated copolymers**							
C8-FASA-based	1315.10/525.90	23	31	1315.10/744.00	8	18	-
C4-FASA-based	1634.20/311.90	10	41	1634.20/219.00	13	26	-

Table S 2. LC gradient method for target PFAS analysis and a separated LC gradient method for the C8- and C4-FASA-based copolymers analysis

**Target PFAS**			**C8- and C4-FASA-based copolymers**		
t (min)	Mobile phase A (%)	Mobile phase B (%)	t (min)	Mobile phase A (%)	Mobile phase B (%)
0.00	99	1	0.00	99	1
0.57	99	1	0.57	99	1
13.00	0	100	2.00	50	50
14.00	0	100	8.00	0	100
14.20	99	1	15..00	0	100
17.00	99	1	15.20	99	1
			17.00	99	1

Flow rate 0.3 ml/min

Table S 3. Instrumental settings for the three different LC-MS/MS methods used to analyze target PFAS (40% and 80% corresponds to the percentage methanol in the extract)

**UPLC-TQ-S MS/MS**	**Target PFAS 40%***	**Target PFAS 80%****	**C8- and C4-FASA-based copolymers**
**Ionisation source:**	ESI operating in negative mode	ESI operating in negative mode	ESI operating in negative mode
**Source temperature:**	150°C	150°C	150°C
**Desolvation temp:**	400°C	200°C	400°C
**Desolvation gas flow:**	800 L/h	800 L/h	800 L/h
**Cone gas flow:**	150 L/h	150 L/h	150 L/h
**Capillary:**	0.70 kV	2.90 kV	3.00 kV

* = target PFAS 40% includes the following classes perfluorosulfonamides, PFSAs, PFCAs, fluorotelomer sulfonic acids, fluorotelomer carboxylic acids, fluorotelomer unsaturated carboxylic acids, PFPiAs, PFPAs, novel compounds

** = target PFAS 80% includes the following classes perfluorosulfonamidoacetic acids, perfluorooctane sulfonamide phosphate diester, polyfluoroalkyl phosphate diesters

Quality Assurance and Quality Control

The five-point external calibration curve for analysis using CIC were in a range of 50 to 1000 ng/mL F and the linearity of the external calibration curve was R^2>0.9999. Since fluoride exist within the CIC system and/or in the surrounding environment, multiple instrumental blanks (empty combustion boat) were conducted before sample analysis to obtain a stable fluoride background signal (relative standard deviation, RSD <5%). Additionally, instrumental blanks were conducted between sample analysis to minimize the carry-over effect. Before quantification, the instrumental blank response was subtracted from the sample response.

Several QC standard injections (PFOA; 250 ng/mL F) were obtained throughout the whole analysis and resulted in a deviation of 7%. Sludge samples were extracted in triplicates within an extraction batch and between extraction days to see the repeatability. The RSD value for EOF was approximately 14% within the extraction batch, whereas between extraction days the RSD value was up to 25%. For the TF analysis, the QC standard injections resulted in a deviation of 5%, and triplicates of sludge samples gave a value of 2.3%.

Several preemptive measures were conducted to obtain a low LOD, such as using PFAS-free consumables and glassware burnt at 450°C for 12 hours. Each extraction batch contained a procedure blank that was used to obtain a LOD. The LOD was determined by the average EOF signal plus three times the SD of all procedure blanks. If EOF contamination was present in the procedure blank, the blank concentration was subtracted before processing further.

Influence of sample treatment on EOF

Table S 4. Extraction efficiency (%) and ion enhancement (+%) or suppression(-%) of mass-labelled standards spiked to a procedure blank and sludge samples (US and SWE) prior to- and after extraction, using three sample pre-treatments: ion pair extraction (IPE), solid phase extraction (SPE) and hexane:acetone extraction (Hx:Ac)

	**IPE**			**SPE**			**Hx:Ac**		
	**No matrix**	**US** (n=3)	**SWE** (n=3)	**No matrix**	**US** (n=3)	**SWE** (n=3)	**No matrix**	**US** (n=3)	**SWE** (n=3)
**Analyte**	Extraction efficiency (ion enhancement, + or suppression, -)								
**Perfluoroalkyl carboxylic acids** (PFCAs)									
$^{13}C_2$ TFA*	71	71 (-76)	68 (-28)	15	52 (-80)	3 (-11)	n.d.	n.d. (-1.7)	n.d. (-10)
$^{13}C_4$ PFBA	74	84 (-7.9)	77 (-1.6)	84	78 (-11)	71 (-0.4)	n.d.	n.d (-7.8)	n.d. (-4.3)
$^{13}C_3$ PFPeA	77	84 (-6.7)	76 (-4.8)	84	89 (-8.8)	83 (-3.7)	n.d.	n.d. (-6.8)	n.d. (-3.4)
$^{13}C_2$ PFHxA	76	85 (-5.7)	79 (-5.1)	86	91 (-8.9)	84 (-3.4)	n.d.	n.d.(-7.7)	n.d. (-2.9)
$^{13}C_4$ PFHpA*	83	46 (-3.8)	79 (+19)	85	87 (10)	78 (+22)	n.d.	n.d (- 8.0).	n.d. (-8.0)
$^{13}C_4$ PFOA	80	88 (-4.2)	79 (-2.2)	87	91 (-7.0)	83 (-1.2)	n.d.	n.d. (- 9.0)	n.d. (-5.7)
$^{13}C_5$ PFNA	80	88(-4.6)	80 (-4.7)	88	90 (-6.9)	80 (-0.8)	n.d.	n.d. (- 8.7)	n.d. (-7.1)
$^{13}C_2$ PFDA	81	86 (-4.4)	77 (-1.9)	81	89 (-4.0)	81 (-4.0)	n.d.	n.d. (- 7.2)	n.d. (-3.5)
$^{13}C_2$ PFUnDA	85	87 (-6.5)	80 (-5.3)	77	88 (-7.6)	79 (-2.1)	n.d.	n.d. (-7.6)	n.d. (-4.8)
$^{13}C_2$ PFTDA*	132	88 (-35)	96 (-3.8)	98	67 (-8.8)	62 (-37)	n.d.	n.d. (-9.3)	n.d. (-29)
$^{13}C_2$ PFDoDA*	94	105 (-43)	87 (92)	80	92 (-30)	93 (+3.3)	n.d.	n.d. (-2.4)	n.d. (-6.2)
$^{13}C_2$ PFHxDA*	106	91 (+370)	94 (410)	127	65 (+560)	71(+200)	n.d.	n.d. (+25)	n.d. (+18)
**Precursors to perfluoroalkyl carboxylic acids**									
$^{13}C_2$ 4:2 FTSA*	81	128 (-37)	77 (-43)	90	85 (+51)	84 (+28)	n.d.	n.d. (- 5.0)	n.d. (-1.7)
$^{13}C_2$ 6:2 FTSA*	85	52 (+60)	82 (+64)	74	87 (+51)	78 (+69)	n.d.	n.d. (- 19)	n.d. (-18)
$^{13}C_2$ 8:2 FTSA*	77	74 (+42)	80 (+84)	63	92 (+31)	84 (+49)	n.d.	n.d. (+ 7.2)	n.d. (-3.1)
$^{13}C_2$ 6:2 FTUCA*	74	69 (+8.4)	80 (-17)	50	66 (+36)	66 (+45)	n.d.	n.d. (-23)	n.d. (-25)
$^{13}C_2$ 8:2 FTUCA*	48	68 (-32)	84 (+68)	51	100 (+64)	80 (+3.7)	n.d.	n.d. (-6.8)	n.d. (-5.1)
$^{13}C_2$ 10:2 FTUCA*	61	90 (-55)	87 (-63)	42	87 (-52)	63 (-20)	n.d.	n.d. (-21)	n.d. (-22)
$^{13}C_2$ 6:2monoPAP*	n.d.	n.d.(-14)	n.d.(+9.4)	n.d.	n.d.(+28)	n.d.(+18)	n.d.	n.d.(-31)	n.d. (-23)
$^{13}C_2$ 8:2monoPAP*	n.d.	n.d.(+44)	n.d. (+50)	n.d.	n.d.(+24)	n.d.(+24)	n.d.	n.d.(-38)	n.d. (-30)

$^{13}C_4$ 6:2 diPAP*	93	87 (+400)	82 (+500)	73	85 (+380)	62(+430)	n.d.	n.d.(+30)	n.d. (+35)
$^{13}C_4$ 8:2 diPAP*	79	98 (+300)	88 (+330)	78	80 (+390)	62(+300)	n.d.	n.d.(-6.8)	n.d. (-4.6)
**Perfluoroalkyl sulfonic acids** (PFSAs)									
$^{13}C_3$ PFBS*	33	110 (-29)	80 (-68)	78	83(+0.8)	80 (-0.5)	87	95 (-15)	85 (-14)
$^{18}O_2$ PFHxS	78	88 (-7.9)	80 (-5.8)	84	91(-8.8)	82 (-6.4)	84	88 (- 9.0)	87 (-8.8)
$^{13}C_4$ PFOS	86	89 (-4.2)	84 (-5.6)	78	92 (-7.0)	83 (-2.2)	93	90 (- 4.2)	80 (-0.2)
**Precursors to perfluoroalkyl sulfonic acids**									
$^{13}C_8$ FOSA*	79	97 (-28)	93 (-30)	67	100 (-21)	142 (-78)	12	2(- 4.9)	1 (-8.0)
$^{2}H_3$ MeFOSA*	n.d.	n.d.	n.d.	n.d.	n.d.	n.d.	n.d.	n.d.	n.d.
$^{2}H_5$ EtFOSA*	n.d.	n.d.	n.d.	n.d.	n.d.	n.d.	n.d.	n.d.	n.d.
$^{2}H_3$ MeFOSAA*	80	81 (+220)	82 (+330)	63	88 (+200)	69(+300)	n.d.	n.d.(-2.9)	n.d. (-0.2)
$^{2}H_5$ EtFOSAA*	80	84 (+160)	83 (+180)	58	87 (+160)	60(+210)	n.d.	n.d.(-1.5)	n.d.(+0.6)

Target PFAS levels used to determine how different sample treatments influence EOF

Table S 5. Average concentrations* (ng/g d.w.) together with the relative standard deviation (%) of target PFAS in sludge samples (US and SWE) using three sample pre-treatments: hexane:acetone extraction (Hx:Ac), solid phase extraction (SPE), and ion pair extraction (IPE). (Only analytes and levels above the detection limit are included in the table).

		**Hx:Ac**		**SPE**		**IPE**	
		**US** (*n*=3)	**SWE** (*n*=3)	**US** (*n*=3)	**SWE** (*n*=3)	**US** (*n*=3)	**SWE** (*n*=3)
**Compound**	**LOD**	Average concentrations ng/g (RSD %)					
**Perfluoroalkyl carboxylic acids** (PFCAs)							
TFA	1.2						
PFPrA	1.2			9.4 (20)		10 (11)	
PFBA	0.08			5.6 (17)		5.0 (3.4)	
PFPeA	0.08			7.0 (1.7)	1.1 (3.6)	5.8 (7.6)	0.5 (15)
PFHxA	0.08			12 (5.9)	2.2 (3.5)	11 (1.9)	1.7 (4.9)
PFHpA	0.08			14 (7)	1.1 (3.7)	12 (3.6)	0.91 (13)
PFOA	0.08			29 (3.2)	11 (0.69)	25 (2.5)	7.6 (6.2)
PFNA	0.08			2.6 (11)	4.6 (9.0)	2.2 (3.5)	4.7 (3.9)
PFDA	0.08			4.7 (7.3)	16 (12)	4.4 (1.0)	14 (0.95)
PFUnDA	0.1			2.5 (15)	21 (18)	2.5 (5.9)	20 (1.5)
PFDoDA	0.2			2.3 (19)	4.4 (19)	2.2 (3.5)	5.2 (1.1)
PFTrDA	0.2			0.59 (12)	2.9 (19)	1.4 (25)	3.9 (9.5)
PFTDA	0.2			0.6 (18)	0.68 (9.1)	0.78 (12)	1.3 (6.4)
PFHxDA	0.8			<0.8	<0.8	<0.8	<0.8
PFOcDA	0.2			1.4 (3.9)	0.36 (18)	1.9 (13)	0.59 (13)
**Precursors to perfluoroalkyl carboxylic acids**							
4:2 FTSA	0.2						
6:2 FTSA	0.2			2.5 (7.0)	0.93 (5.1)	1.8 (5.0)	0.74 (7.2)
8:2 FTSA	0.2			4.4 (8.0)	3.7 (8.4)	4.1 (2.6)	3.9 (3.0)
10:2 FTSA	0.5				3.8 (11)		5.7 (15)
3:3 FTCA	0.5						
5:3 FTCA	0.5			18 (6.7)	51 (5.5)	17 (25)	54 (2.1)
7:3 FTCA	0.5			26 (15)	82 (5.3)	18 (8.4)	160 (12)
6:2 FTUCA	0.5				4.3 (4.6)		2.6 (3.8)
8:2 FTUCA	0.5				9.7 (6.0)		7.9 (4.6)
10:2 FTUCA	0.5				0.62 (20)		0.52 (2.2)
6:2 monoPAP	1.2	n.d.	n.d.	n.d.	n.d.	n.d.	n.d.
8:2 monoPAP	1.2	n.d.	n.d.	n.d.	n.d.	n.d.	n.d.
10:2 monoPAP	1.2	n.d.	n.d.	n.d.	n.d.	n.d.	n.d.
4:2 diPAP	1.0						
4:2/6:2 diPAP	1.0						
2:2/8:2 diPAP	1.0						
6:2 diPAP	0.5			11.5 (5.7)	2.4 (5.8)	13 (0.73)	5.0 (5.6)
4:2/8:2 diPAP	0.5						
2:2/10:2 diPAP	0.5						
8:2 diPAP	0.5			15 (8.1)	7.8 (5.6)	21 (3.2)	17 (4.7)

Compound	LOD	IPE		SPE		Hx:Ac	
		US (*n*=3)	SWE (*n*=3)	US (*n*=3)	SWE (*n*=3)	US (*n*=3)	SWE (*n*=3)
**Precursors to perfluoroalkyl carboxylic acids** (PFCAs)							
6:2/10:2 diPAP	0.5			16 (7.5)	4.7 (2.2)	26 (5.3)	11 (4.1)
4:2/12:2 diPAP	0.5						
6:2/8:2 diPAP	0.5			28 (8.7)	5.3 (6.1)	35 (3.3)	10 (2.0)
4:2/10:2 diPAP	0.5						
8:2/10:2 diPAP	1.0			31 (14)	18 (7.7)	50 (3.3)	41 (2.6)
6:2/12:2 diPAP	1.0			15 (13)	5.3 (4.0)	23 (4.6)	11 (7.0)
10:2 diPAP	1.0			10 (17)	4.7 (13)	14 (1.2)	11 (5.0)
8:2/12:2 diPAP	1.0			17 (14)	8.9 (13)	22 (5.6)	20 (2.5)
6:2/14:2 diPAP	1.0			7.1 (11)	2.6 (5.8)	7.9 (5.7)	5.7 (6.5)
10:2/12:2 diPAP	1.0			5.1 (12)	2.1 (18)	6.0 (6.5)	4.8 (2.3)
8:2/14:2 diPAP	1.0			3.6 (10)	2.1 (9.5)	3.8 (7.0)	4.0 (9.2)
12:2 diPAP	1.0						
10:2/14:2 diPAP	1.0						
8:2/16:2 diPAP	1.0						
**Perfluoroalkyl sulfonic acids** (PFSAs)							
TFMS	1.2						
PFEtS	0.8						
PFPrS	0.8						
PFBS	0.08						
PFPeS	0.08						
PFHxS	0.08	5.7 (5.2)		13 (5.6)			
PFHpS	0.08	0.62 (12)		0.67 (3.8)			
PFOS	0.08	147 (4.3)	8.5 (4.0)	178 (3.6)	16 (10)	164 (3.4)	16 (3.9)
PFNS	0.08	5.3 (5.1)		6.3 (6.9)		4.9 (1.5)	
PFDS	0.1	146 (5.9)	1.0 (13)	121 (3.6)		105 (1.4)	
PFDoDS	0.4						
**Precursors to perfluoroalkyl sulfonic acids**							
FBSA	0.2						
MeFBSA	0.2			3.8 (17)		3.6 (25)	
FHxSA	0.5			<0.5			
MeFHxSA	0.2						
FOSA	0.08			5.2 (5.5)	1.6 (17)	5.0 (1.3)	2.0 (3.9)
MeFOSA	0.2	n.d.	n.d.	n.d.	n.d.	n.d.	n.d.
EtFOSA	0.2	n.d.	n.d.	n.d.	n.d.	n.d.	n.d.
FOSAA	0.2			14 (11)	2.6 (0.68)	14 (2.8)	3.9 (4.6)
MeFOSAA	0.2			41 (5.1)	8.5 (6.6)	45 (0.39)	11 (0.75)
EtFOSAA	0.2			290 (6.7)	15 (5.9)	317 (1.4)	22 (0.88)
SAmPAP	1.0	n.d.	n.d.	n.d.	n.d.	n.d.	n.d.
diSAmPAP	0.5			67 (9.5)	0.51 (20)	99 (5.8)	0.73 (3.5)
**Perfluoroalkyl phosphonic acids** (PFPAs)							
PFHxPA	0.1				0.60 (6.0)		0.59 (6.5)
PFDPA	0.1				41 (14)		93 (24)
PFOPA	0.1				8.9 (12)		7.2 (25)

Compound	LOD	IPE US (*n*=3)	IPE SWE (*n*=3)	SPE US (*n*=3)	SPE SWE (*n*=3)	Hx:Ac US (*n*=3)	Hx:Ac SWE (*n*=3)
**Perfluoroalkyl phosphinic acids** (PFPiAs)							
6:6 PFPiA	0.5		18 (3.0)	1.7 (20)	37 (16)	2.8 (24)	58 (21)
6:8 PFPiA	0.5	0.95 (20)	80 (8.0)	3.4 (19)	74 (21)	4.7 (22)	333 (25)
8:8 PFPiA	0.5	2.2 (25)	189 (14)	8.2 (6.0)	161 (25)	25 (25)	1108 (22)
**Novel PFAS**							
8:2 Cl-PFESA	1.0						
6:2 Cl-PFESA	1.0						
PFECHS	1.0	1.69 (4.1)		2.8 (5.3)		2.3 (9.0)	
HFPO-DA	1.0						
ADONA	1.2						
**Side-chain fluorinated copolymer**							
C8-FASA-based	20	4340 (25)					
C4-FASA-based	20		480 (10)				
**Fluorinated pharmaceuticals**							
Fluoxetine	1.0	8.7 (2)	11 (2)	10 (10)	33 (17)		
Celecoxib	2.0		82 (3)		72 (13)		160 (3)

*Concentrations were only used for the fluorine mass balance analysis. The levels do not represent the actual concentration in the sample itself, because they were not recovery corrected

Target PFAS levels in sludge used for the fluorine mass balance analysis

Table S 6. Individual PFAS concentrations (ng/g d.w., not recovery corrected) for fluorine mass balance analysis in sewage sludge samples collected between 2004 and 2017 from two WWTPs (Borås and Stockholm) using the hexane:acetone extraction. Year 2006 for both sites is not included and 2005 is not included for Borås WWTP. (Only analytes and concentrations above the detect limit are included in the table)

Hexane:Acetone Extraction

Sludge

	**Borås WWTP**														**Umeå WWTP**	**Torsås WWTP**
	**LOD**	**2004**	**2015**	**2015**	**2008**	**2009**	**2010**	**2011**	**2012**	**2013**	**2014**	**2015**	**2016**	**2017**	**2015**	**2015**
**PFHxS**	0.04	0.13	N/A*	0.06	0.10	0.11	0.08	0.09	0.04	0.07	0.08	0.05			0.04	0.06
**PFHpS**	0.04		N/A					0.04								
**PFOS**	0.08	6.3	N/A	3.9	7.1	3.6	3.5	4.4	2.8	2.0	2.2	2.9	0.14	0.13	1.7	0.90
**PFDS**	0.04	0.66	N/A	0.20	0.17	0.12	0.09		0.07							
**6:2 FTSA**	0.10	0.83	N/A		0.46						1.4	0.97				
**FOSA**	0.06	2.6	N/A	2.2	0.80	0.83	0.81	0.68	0.67	0.16	0.61	0.7			0.06	0.11
**6:6PFPiA**	0.10	46	N/A	0.77	0.33	0.34	0.27	0.36	0.24	0.29		0.15				
**6:8PFPiA**	7.9	190	N/A													
**8:8PFPiA**	19	600	N/A													

	**Stockholm WWTP**															
	**LOD**	**2004**	**2005**	**2007**	**2008**	**2009**	**2010**	**2011**	**2012**	**2013**	**2014**	**2015**	**2016**	**2017**		
**PFOS**	0.04	0.49	0.47	0.34	0.28	0.41	0.26	0.24	0.24	0.15	0.11	0.09	0.04	0.11		
**PFDS**	0.04	0.1	0.04													
**6:6PFPiA**	0.10		0.11													

Note: Concentrations were only used for the fluorine mass balance analysis. The levels do not represent the actual concentration in the sample itself, because the method used was not optimized for these PFASs, it only represent the known fluorine in the extractable organofluorine.

* N/A = not available

Table S 7 Individual PFAS concentrations (ng/g d.w., not recovery corrected) for fluorine mass balance analysis in sewage sludge samples collected between 2004 and 2017 from two WWTPs (Borås and Stockholm) using the ion pair extraction. Year 2006 for both sites is not included and 2005 is not included for Borås WWTP. (Only analytes and concentrations above the detect limit are included in the table)

Sludge	Ion Pair Extraction															
	Borås WWTP														Umeå WWTP	Torsås WWTP
	LOD	2004	2005	2007	2008	2009	2010	2011	2012	2013	2014	2015	2016	2017	2015	2015
PFBA	0.1															4.5
PFHxA	0.2	1.8	2.5	0.7	1.7	4.0	5.6	1.3	0.7	1.1	2.1	1.9	N/A*	N/A*		
PFHpA	0.5	1.2		0.6						0.5			N/A	N/A		
PFHxS	0.2	0.4		0.2			2.0			0.3			N/A	N/A		
PFHpS	0.1							0.1					N/A	N/A		
PFOA	0.3	11.5	17.4	4.1	7.7	22.2	15.2	2.9	2.3	2.3	1.6	1.4	N/A	N/A	0.3	0.9
PFNA	0.4	8.8	14.2	3.4	3.7	3.0	1.7	0.6	0.5	2.5	0.4		N/A	N/A		
PFOSA	0.1	2.3	1.4	0.8	0.5	0.5	0.6	0.4		0.5	0.3	0.4	N/A	N/A	0.1	
PFOS	2.9	16.6	7.9	6.9	11.8	8.4	9.7	11.9	6.8	7.1	4.0	4.6	N/A	N/A	4.3	5.7
PFDA	0.5	17.6	27.0	11.8	11.0	19.5	19.4	4.8	4.1	6.7	2.6	1.8	N/A	N/A	0.4	1.3
PFUnDA	0.6	34.5	47.3	24.6	34.4	22.4	13.2	3.7	2.2	9.0	1.5	1.1	N/A	N/A	0.3	0.7
PFDS	0.1	1.0	2.3	0.2	0.6	1.9	0.8						N/A	N/A		
PFDoDA	0.1	6.9	8.1	2.9	4.4	2.1	1.9	1.5	1.4	1.4	0.7	0.6	N/A	N/A	0.5	0.1
PFTrDA	0.1	5.3	12.1	3.0	12.4	4.2	1.4		0.6	1.3	0.7	0.2	N/A	N/A	0.1	
PFTDA	0.1	1.4	1.4	0.6	0.8	0.5	0.4			0.3	0.2	0.2	N/A	N/A	0.2	
PFHxDA	0.1	0.6	0.4	0.2	0.2	0.2	0.2		0.2	0.3	0.1	0.1	N/A	N/A	0.2	
PFOcDA	0.1	0.1	0.1		0.1	0.1	0.1			0.1			N/A	N/A	0.1	
4:2 FTSA	0.1						1.2	0.1					N/A	N/A		
6:2 FTSA	0.1	0.1	4.2	0.2	0.2	0.6	0.2	0.1	0.4	0.5	2.0	0.6	N/A	N/A	0.2	
8:2 FTSA	0.1	3.9	3.5	1.6	1.7	2.0	1.7	1.0	0.8	1.3	0.9	0.7	N/A	N/A	0.8	0.8
5:3 FTCA	0.1	5.1	19.5	7.0	33.9	88.4	127.9	177.4	33.1	128.9	266.6	279.9	N/A	N/A	6.4	7.8
7:3 FTCA	0.1	76.3	219.9	34.4	117.6	150.3	101.3	10.5	12.0	32.7	13.2	12.9	N/A	N/A	2.5	3.9
8:2 FTUCA	0.1	0.1			0.1			0.1	0.1	0.1	0.2	0.2	N/A	N/A	0.1	0.1
MeFOSE	0.1	8.9	18.9	5.5	4.3	6.0	7.5	3.0	3.5	1.6	3.3	4.6	N/A	N/A		3.8
EtFOSE	0.1	37.4	43.8	16.7	8.7	9.6	10.7	9.1	11.6	5.0	4.3	7.0	N/A	N/A		7.4
FOSAA	0.1	1.0		0.6				0.3	0.3		0.1		N/A	N/A		
MeFOSAA	0.1	20.7	46.4	6.6	4.8	10.4	13.4	3.7	3.9	1.2	1.0	4.1	N/A	N/A	0.4	0.6
EtFOSAA	0.1	22.8	37.8	11.0	5.8	11.8	11.3	4.4	6.4	0.6	1.2	5.3	N/A	N/A	2.9	4.4

diSAmPAP	0.1	1.2	1.8	0.3	0.2	0.5	0.3	0.2	0.1			0.1	N/A	N/A		
6:2 diPAP	0.3	2.3	5.9	2.7	0.6	3.2	6.4	3.0	1.9	0.5	0.3	3.3	N/A	N/A	2.9	0.5
8:2 diPAP	0.1	4.5	19.2	9.7	3.2	24.8	25.9	3.9	2.4	0.4	0.4	4.4	N/A	N/A	1.8	1.0
6:2/10:2 diPAP	0.1	5.8	8.2	7.4	2.2	5.1	8.3	6.0	4.1	0.7	0.6	5.9	N/A	N/A	2.5	1.5
6:2/8:2 diPAP	0.1	5.6	13.6	7.1	2.2	8.7	14.1	6.4	4.0	0.7	0.5	4.2	N/A	N/A	3.0	1.7
8:2/10:2 diPAP	0.1	10.1	11.1	18.7	6.6	11.6	10.4	7.1	6.3	0.8	0.7	8.2	N/A	N/A	4.2	1.6
6:2/12:2 diPAP	0.1	5.1	11.3	6.4	3.1	9.9	12.2	4.6	3.7	0.5	0.5	4.6	N/A	N/A	2.9	0.9
10:2 diPAP	0.1	17.6	72.2	26.0	11.3	65.2	79.5	15.7	11.9	1.6	1.5	14.5	N/A	N/A	9.4	3.0
8:2/12:2 diPAP	0.1	30.4	32.6	56.8	18.8	26.4	34.1	27.5	21.7	3.0	1.9	29.9	N/A	N/A	14.9	5.7
6:2/14:2 diPAP	0.1	10.8	10.0	10.2	2.7	5.3	11.7	9.4	8.8	1.0	1.2	11.8	N/A	N/A	5.9	1.7
10:2/12:2 diPAP	0.1	6.6	8.4	11.8	4.4	6.6	9.5	6.7	5.8	0.8	1.0	9.4	N/A	N/A	5.7	1.0
8:2/14:2 diPAP	0.1	6.4	7.1	9.4	3.5	6.8	9.1	4.6	3.9	0.6	0.3	7.8	N/A	N/A	3.5	0.4
12:2 diPAP	0.1	0.2	4.6	0.4	0.1	4.9	5.7	4.3	0.1		0.6	7.3	N/A	N/A	2.5	
10:2/14:2 diPAP	0.1	1.3	0.9	1.2	0.8	1.3	1.7	1.2	1.1	0.1	0.1	2.3	N/A	N/A	0.7	0.2
8:2/16:2 diPAP	0.1	0.9	0.9	1.4	0.5	1.1	1.2	1.0	0.8	0.1	0.1	1.0	N/A	N/A	0.4	
PFOPA	0.1	304.5											N/A	N/A		
PFDPA	0.1	52.4	71.3										N/A	N/A		
6:6 PFPiA	0.1	0.3											N/A	N/A		

Stockholm WWTP

	LOD	2004	2005	2007	2008	2009	2010	2011	2012	2013	2014	2015	2016	2017		
**PFHxA**	0.3	0.7	1.2	0.8	1.0	0.8	0.8	0.6	0.5	0.6	0.5	0.7	N/A	N/A		
**PFHpA**	0.1		0.1	0.2	0.3		0.1	0.1				0.2	N/A	N/A		
**PFHxS**	0.3	1.3	1.4	0.9	1.2	1.0	0.8	1.3	1.3	1.3	1.2	1.4	N/A	N/A		
**PFHpS**	0.1	0.1									0.1		N/A	N/A		
**PFOA**	0.3	1.0	1.1	0.9	1.7	0.9	1.0	0.7	0.5	0.5	0.4	0.5	N/A	N/A		
**PFNA**	0.1	0.1	0.2	0.4	0.2	0.2	0.2	0.3	0.3	0.2	0.2	0.2	N/A	N/A		
**PFOSA**	0.3	0.7	1.2	0.7	0.6	0.7	0.7	0.6	0.6	0.4	0.4	0.3	N/A	N/A		
**PFOS**	2.9	20.2	17.8	15.1	11.2	12.0	7.6	7.1	11.1	6.1	5.1	4.9	N/A	N/A		
**PFDA**	0.5	2.4	3.2	2.4	2.7	2.3	2.8	2.4	2.4	1.9	2.1	2.5	N/A	N/A		
**PFUnDA**	0.6	0.7	0.7	0.8	0.8	0.7	0.8	0.9	0.8	0.7	0.7	0.7	N/A	N/A		
**PFDS**	0.1	4.9	4.8	2.2	1.8	1.0	1.0	0.6	0.5	0.6	0.5	0.4	N/A	N/A		
**PFDoDA**	0.9	1.7	1.9	1.4	1.3	1.0	1.4	1.3								
**PFTrDA**	0.2	0.3	0.3	0.3	0.4	0.3	0.3	0.3	0.3	0.3	0.2	0.3	N/A	N/A		
**PFTDA**	0.3	0.5	0.5	0.5	0.4	0.4	0.4	0.5	0.5	0.4	0.3	0.4	N/A	N/A		
**PFHxDA**	0.3	0.3	0.3	0.3	0.3	0.3	0.3	0.3	0.3	0.3	0.3	0.3	N/A	N/A		
**6:2 FTSA**	0.1	0.2	0.2	0.3	0.5	0.3	0.2	0.2	0.3	0.2	0.1	0.2	N/A	N/A		
**8:2 FTSA**	0.1	2.3	2.4	1.8	2.1	2.0	1.4	1.7	1.6	1.0	1.1	0.9	N/A	N/A		
**5:3 FTCA**	0.1	15.0	30.0	25.7	27.3	23.7	24.5	22.2	18.6	19.1	20.7	27.0	N/A	N/A		

**7:3 FTCA**	0.1	24.7	17.1	21.6	41.8	24.7	18.5	25.2	23.0	17.6	19.6	14.8	N/A	N/A	
**8:2 FTUCA**	0.1	0.1		0.1	0.1	0.1		0.2			0.1		N/A	N/A	
**N-MeFOSE**	0.1	2.0	6.6				1.4						N/A	N/A	
**FOSAA**	0.1	0.8	0.7	0.4	0.4	0.4	0.6	0.4	0.2	0.2	0.3	0.3	N/A	N/A	
**N-MeFOSAA**	0.1	4.0	2.9	2.0	1.4	1.3	1.5	1.4	1.4	1.0	1.0	1.1	N/A	N/A	
**N-EtFOSAA**	0.1	10.6	9.8	5.6	4.6	3.7	3.4	3.5	2.4	2.2	2.0	2.3	N/A	N/A	
**diSAmPAP**	0.1	0.2	0.3	0.1	0.1	0.1		0.1	0.1			0.1	N/A	N/A	
**6:2 diPAP**	0.3	0.7	0.6	1.5	0.7	0.6	0.4	1.1	0.4	0.9	0.9	1.2	N/A	N/A	
**4:2/8:2 diPAP**	0.1			0.1									N/A	N/A	
**8:2 diPAP**	0.1	1.6	2.1	4.5	2.6	2.4	1.6	2.6	1.1	1.5	1.1	1.8	N/A	N/A	
**6:2/10:2 diPAP**	0.1	2.1	2.7	5.8	2.5	1.9	2.0	3.2	1.4	1.8	1.4	2.6	N/A	N/A	
**8:2/10:2 diPAP**	0.1	1.6	2.1	4.8	2.0	1.7	1.3	2.6	0.9	1.1	0.8	1.4	N/A	N/A	
**6:2/12:2 diPAP**	0.1	4.0	5.3	10.1	7.4	7.5	4.9	5.0	2.6	2.9	2.1	3.8	N/A	N/A	
**10:2 diPAP**	0.1	1.8	1.9	4.3	2.4	2.4	1.6	2.0	1.1	1.3	1.1	2.5	N/A	N/A	
**8:2/12:2 diPAP**	0.1	8.0	7.6	16.8	10.1	11.1	6.7	6.9	4.3	4.8	4.5	9.9	N/A	N/A	
**6:2/14:2 diPAP**	0.1	19.0	19.9	39.3	25.9	29.0	19.5	19.1	10.5	12.4	9.6	19.6	N/A	N/A	
**10:2/12:2 diPAP**	0.1	6.4	6.5	16.9	6.9	4.9	7.3	9.2	4.1	6.1	4.0	10.0	N/A	N/A	
**8:2/14:2 diPAP**	0.1	5.7	5.8	13.5	8.3	7.6	6.6	7.0	3.0	4.1	3.8	8.9	N/A	N/A	
**12:2 diPAP**	0.1	4.9	5.4	13.3	7.3	8.1	6.1	6.6	2.9	3.5	2.8	5.4	N/A	N/A	
**10:2/14:2 diPAP**	0.1	2.7	3.0	7.1	4.2	4.0	3.4	4.0	1.8	2.1	2.4	4.4	N/A	N/A	
**8:2/16:2 diPAP**	0.1	0.4	1.1	2.5	1.3	1.1	1.3	1.5	0.4	0.8	0.7	1.5	N/A	N/A	
**8:2/10:2 diPAP**	0.1	1.0	1.2	2.8	1.7	1.3	0.9	1.3	0.6	0.6	0.7	1.4	N/A	N/A	
**PFOPA**	0.1	0.5	0.1										N/A	N/A	
**PFDPA**	0.1	1.7	1.0		0.2	0.2	0.1	0.1	0.2	0.2	0.2		N/A	N/A	
**6:6 PFPiA**	0.1	1.2	1.1	0.5	0.3		0.2	0.1	0.4	0.2	0.2	0.1	N/A	N/A	
**6:8 PFPiA**	0.1	4.0	3.6	1.6	0.9		0.8	0.5	1.2	0.4	0.3	0.3	N/A	N/A	
**8:8 PFPiA**	0.1	9.9	9.5	3.8			2.1		3.0				N/A	N/A	

Note: Concentrations were only used for the fluorine mass balance analysis. The levels do not represent the actual concentration in the sample itself, because they were not recovery corrected, it only represent the known fluorine in the extractable organofluorine.

* N/A = not available
